# Synthesis of vinyl esters of aromatic carboxylic acids in the presence of Zn/SiOC, ZnO/SiOC, and Ni/SiOC catalytic systems

**DOI:** 10.55730/1300-0527.3750

**Published:** 2025-08-05

**Authors:** Shavkat TURSUNOV, Askar PARMANOV, Suvankul NURMANOV, Odiljon ZIYADULLAYEV, Dilshod BOYKOBILOV, Liudmila KHOROSHKO, Monika WILAMOWSKA-ZAWLOCKA, Balanand SANTHOSH, Olim RUZIMURADOV

**Affiliations:** 1 Department of Organic and Oil & Gas Chemistry, Faculty of Chemistry, National University of Uzbekistan, Tashkent, Uzbekistan; 2 Department of Chemistry, Faculty of Natural Sciences, Chirchik State Pedagogical University, Uzbekistan; 3 Department of Micro- and Nanoelectronics, Faculty of Radioengineering and Electronics, Belarusian State University of Informatics and Radioelectronics, Minsk, Belarus; 4 R&D Lab of Energy-Efficient Materials and Technologies, Faculty of Physics, Belarusian State University, Minsk, Belarus; 5 Department of Energy Conversion and Storage, Faculty of Chemistry, Gdańsk University of Technology, Gdansk, Poland; 6 Department of Natural-Mathematical Sciences, Turin Polytechnic University in Tashkent, Tashkent, Uzbekistan

**Keywords:** Carboxylic acids, vinylation, Zn/SiOC, vinyl esters

## Abstract

In this work, various aromatic monocarboxylic and dicarboxylic acids were subjected for the first time to a vinylation reaction with acetylene under heterogeneous catalytic conditions using catalytic systems based on silicon oxycarbide supported on silicon carbide (SiC): zinc silicon oxycarbide (Zn/SiOC), zinc oxide silicon oxycarbide (ZnO/SiOC), and nickel silicon oxycarbide (Ni/SiOC). The influence of the nature of the starting materials, temperature, reaction duration, solvent, and catalyst type on the yield of vinyl esters was investigated. The vinylation reaction of aromatic carboxylic acids with acetylene was carried out at 1:2 molar ratio, using a Zn/SiOC-50 catalytic system at a loading of 10 mol% relative to the initial aromatic carboxylic acid, in a N,N-dimethylformamide (DMF) solution at 150 C for 12 h, resulting in high yields of vinyl esters. Under these heterogeneous catalytic conditions, the vinylation reaction afforded the following vinyl esters: benzoic acid (80%), 4-methylbenzoic acid (77%), 4-methoxybenzoic acid (70%), 4-fluorobenzoic acid (83%), 4-tert-butylbenzoic acid (65%), 4-chlorobenzoic acid (85%), divinyl esters of ortho-phthalic acid (88%), and terephthalic acid (91%). The structures of the synthesized vinyl esters were confirmed by Fourier-transform infrared (FTIR), proton nuclear magnetic resonance (^1^H NMR), carbon-13 nuclear magnetic resonance (^13^C NMR), and chromatographic-mass spectral (MS) analyses.

## 1. Introduction

A wide variety of organic compounds, including vinyl esters of aromatic carboxylic acids, are widely utilized in both industrial applications and research laboratories due to their chemical reactivity and broad range of functional properties. Their high reactivity enables them to be used in synthesizing polyolefins and biologically active compounds, which broadens their scope of application even further [1,2].

Vinyl esters containing aromatic rings are currently used frequently in developing highly durable and thermally stable polymeric materials, as active agents in the medical and pharmaceutical fields, and as intermediates in organic synthesis [3]. Furthermore, their high polymerization potential makes them valuable precursors for the fabrication of photosensitive materials, biodegradable polymers, energy storage devices, and emerging technologies, such as green energy systems. These diverse applications highlight the need for further in-depth investigation of such compounds [4–7].

Vinylation of carboxylic acids under heterogeneous catalytic conditions is a key transformation in organic synthesis. This process enables the incorporation of the vinyl group (−CH=CH_2_) into carboxylic acid derivatives. This reaction is typically catalyzed by heterogeneous systems that contain metals or metal oxides [8].

Vinylation reactions have been reported using heterogeneous catalysts, such as palladium on carbon (Pd/C), copper on carbon (Cu/C), and transition metal oxides like TiO_2_ and ZrO_2_ supported on various materials [9,10]. These reactions are generally carried out at temperatures ranging from 100 to 200°C, depending on the nature of the catalyst and substrates used [11]. The vinylation process can be carried out under atmospheric pressure for 6–24 h or under elevated pressure in an inert atmosphere (e.g., nitrogen or argon). Aprotic solvents, such as dimethyl sulfoxide (DMSO), N,N-dimethylformamide (DMF), and toluene are commonly used. Vinylating agents include vinyl halides (e.g., vinyl chloride and vinyl bromide), vinyl esters, vinyl acetate, and acetylene. They have enabled the obtaining of vinyl esters derived from aliphatic, aromatic, heterocyclic, and other carboxylic acids [12–14].

In this work, acetylene (2) was used as the vinylating agent under heterogeneous catalytic conditions to form vinyl esters (3) from a variety of substituted aromatic carboxylic acids (1) in a novel way. Our work introduces a novel class of inexpensive, thermally stable silicon oxycarbide (SiOC)-based catalysts doped with Zn, ZnO, and Ni, in contrast to earlier research that mostly used homogeneous systems or expensive noble metal catalysts (such as Pd, Pt). Significantly, we demonstrate, for the first time, the efficacy of zinc silicon oxycarbide (Zn/SiOC), zinc oxide silicon oxycarbide (ZnO/SiOC), and nickel silicon oxycarbide (Ni/SiOC) catalytic systems in these types of vinylation reactions. Furthermore, we systematically examined the effect of catalyst loading, reaction temperature, time, solvent, metal type, and metal content affected on product yield. This thorough analysis demonstrates that the Zn/SiOC-50 system has high catalytic activity and is recyclable and potentially scalable for environmentally friendly vinyl ester synthesis.

## 2. Experimental section

### 2.1. Instrumentation and chemicals

All reagents and solvents were used as received without further purification. Poly(methylhydrosiloxane) (PMHS, ≥99% purity), divinylbenzene (DVB, 98%), Karstedts catalyst (Pt ≈ 2 wt%), zinc acetate dihydrate (Zn(CH_3_COO)_2_·2H_2_O, ≥99%), anhydrous zinc acetate (Zn(CH_3_COO)_2_, ≥98%), and nickel(II) acetylacetonate (Ni(acac)_2_, ≥97%) were all purchased from Sigma-Aldrich (Merck KGaA, Darmstadt, Germany). Aromatic carboxylic acids such as benzoic acid (≥99.5%), p-toluic acid (≥98%), and p-chlorobenzoic acid (≥98%) were also obtained from Sigma-Aldrich and used without further purification. Acetylene (C_2_H_2_), with a minimum purity of 99.5%, was obtained in pressurized cylinders and handled under well-ventilated fume hoods with appropriate gas regulators and flow controllers. Fourier-transform infrared (FTIR) spectrums of the synthesized compounds were recorded on Bruker Furye Invenio S-2021 (Bruker Corp., Billerica, Massachusetts, U.S.) spectrometer in the range of 4000–400 cm^−1^. ^1^H NMR spectrums were obtained at on a Varian Unity+ 400 MHz (Agilent Technologies, Santa Clara, California, U.S.) instrument using CD_3_OD, CDCl_3_, or pyridine-d_5_ as solvents. Hexamethyldisilazane was used as an internal standard for the ^1^H NMR spectra, while the residual solvent peak was used as the reference for the ^13^C NMR spectra. Mass spectra were acquired using an 7890B Network GC system chromatograph–mass spectrometer (Agilent Technologies, Santa Clara, California, U.S.). Raman spectroscopy was performed on a SENTERRA II micro-Raman spectrometer (Horiba Jobin Yvon, Bensheim, Germany) equipped with an argon laser (λ = 514.5 nm). X-ray diffraction (XRD) patterns were collected on a MiniFlex 600 X-ray diffractometer (Rigaku Holdings Corporation, Tokyo, Japan) in flat sample transmission geometry using Cu K_α_ radiation. Scans were conducted at a speed of 1 scan per min over a 10–50°C range for a total duration of 2 h.

### 2.2. Synthesis of metal-loaded silicon oxycarbide (Me/SiOC) catalytic systems

The catalytic systems based on silicon oxycarbide were synthesized according to the methods adopted from literature [15–17]. The synthesis of silicon oxycarbide-based catalysts (Zn/SiOC, ZnO/SiOC, and Ni/SiOC) was conducted as follows. The hydrosilylation reaction was started by dissolving 3.0 g of poly(methylhydrosiloxane), 6.0 g of divinylbenzene, and 5 μL of Karstedts catalyst (platinum(0)-1,3-divinyl-1,1,3,3-tetramethyldisiloxane) in 10 mL of isopropanol in a 25 mL flat-bottom flask. The mixture was then mechanically stirred for 30 min at room temperature. Zinc acetate dihydrate (Zn(CH_3_COO)_2_·2H_2_O) was added in proportions of 10%, 30%, 50%, and 70% (weights of 1.0 g, 3.85 g, 9.0 g, and 21.0 g, respectively) metal loading to prepare Zn/SiOC. Anhydrous zinc acetate (Zn(CH_3_COO)_2_, 21.0 g) was utilized as the precursor for the ZnO/SiOC synthesis. Nickel (II) bis(acetylacetonate) (C_10_H_14_NiO_4_) was added in comparable amounts (1.0 g, 3.85 g, 9.0 g, or 21.0 g) for Ni/SiOC. After stirring the mixtures for another 30 min at room temperature, gelation took place in about 3 h. The resultant preceramic gels were dried for 48 h at 80 C in a standard oven. The dried gels were then heated at a rate of 2 C/min to 1000 C in a quartz tube furnace with an argon atmosphere (30 mL/min), held there for 90 min, and then cooled to room temperature at the same rate. The resultant black ceramic materials were utilized as heterogeneous catalysts in the vinylation reactions after being finely powdered. The characteristics of the synthesized catalysts were investigated by XRD and Raman spectroscopy.

### 2.3. Vinylation reaction of aromatic carboxylic acids with acetylene under heterogeneous catalytic conditions

The vinylation reactions were carried out in a selected autoclave. The catalytic systems based on silicon oxycarbide (SiOC) supported on silicon carbide (SiC) were used at a loading of 10 mol% relative to the initial amount of aromatic carboxylic acid (0.112 g) using Zn/SiOC, ZnO/SiOC, and Ni/SiOC catalysts. Substituted aromatic carboxylic acids (10 mmol) were dissolved in 10 mL of DMFA and introduced into the reactor. Acetylene (20 mmol, approximately 450 mL) was added from gas cylinder. The reaction mixture was heated at 150 C for 12 h under inert argon atmosphere (225 mL). After completion, the reaction mixture was cooled to room temperature. To remove any metal ions leached into the solution, 0.5 g of activated carbon was added and stirred for 30 min, followed by filtration. The filtrate was extracted three times with diethyl ether (3 × 15 mL), then sequentially washed with 0.5 M NaHCO_3_, 1 M NaHSO_4_, distilled water, and a saturated potassium chloride solution. The organic layer was dried over 5 g of Na_2_SO_4_ for 12 h and filtered. The solvent was evaporated under vacuum, and the residue was dried in a vacuum desiccator over calcium chloride (CaCl_2_) at room temperature until a constant mass was achieved. The obtained 3a–h were analyzed by FTIR, ^1^H NMR, ^13^C NMR, and chromatographic-mass spectral analyses. The corresponding spectral data of the obtained 3a–h are presented below.

#### Vinyl ester of benzoic acid (3a)

Yield: 80%; colorless oily liquid; Bp: 66–67°C / 50 mmHg; Rf = 0.65. FTIR (cm^−1^): 3091 (Ar–H), 1728 (C=O), 1645 (CH=CH_2_), 1601 (Ar C=C), 1452 (CH_2_ deformation), 1246 (C–O–C). ^1^H NMR (400 MHz, CD_3_OD, δ, ppm): 8.00 (2H, d, J = 9.7 Hz, Ar-H, C-2,6), 7.55 (1H, t, J = 5.9 Hz, −CH=), 7.41–7.42 (4H, m, Ar-H, C-3,4,5), 5.03 (1H, dd, J = 14.2, 2.0 Hz, =CH_2_), 4.65 (1H, dd, J = 6.4, 1.5 Hz, =CH_2_). ^13^C NMR (CD_3_OD, δ, ppm): 164.6 (C=O), 142.4 (C–O–C), 134.7 (C-4), 130.7 (C-3,5), 129.6 (C-2,6), 98.6 (=CH_2_). LC–MS: m/z calcd. for C_9_H_8_O_2_^+^: 148.167; found: 148.8.

#### Vinyl ester of 4-methylbenzoic acid (3b)

Yield: 77%; colorless oily liquid; Bp: 72–73°C / 50 mmHg; Rf = 0.70. FTIR (cm^−1^): 1729 (C=O), 1646 (CH=CH_2_). ^1^H NMR (400 MHz, CD_3_OD, δ, ppm): 8.00 (2H, d, J = 8.3 Hz, Ar-H), 7.55 (2H, d, J = 7.9 Hz, Ar-H), 7.45 (1H, dd, J = 14.2, 6.4 Hz, −CH=), 4.80 (1H, dd, J = 13.6, 1.4 Hz, =CH_2_), 4.50 (1H, dd, J = 6.3, 1.4 Hz, =CH_2_), 1.43 (3H, s, CH_3_). ^13^C NMR (CD_3_OD, δ, ppm): 172.6 (C=O), 144.3, 141.4 (vinyl C), 139.2 (C-4), 131.3 (C-2,6), 129.2 (C-3,5), 95.1 (=CH_2_), 31.6 (CH_3_).

#### Vinyl ester of 4-methoxybenzoic acid (3c)

Yield: 70%; white crystalline solid; Bp: 59–61°C; Rf = 0.45. FTIR (cm^−1^): 2975 (CH_3_), 1724 (C=O), 1645 (CH=CH_2_), 1607 (Ar C=C), 1425 (=CH), 1261 (C–O–C). ^1^H NMR (400 MHz, CDCl_3_, δ, ppm): 3.76–3.64 (1H, dd, trans-CH_2_), 4.02 (3H, s, −OCH_3_), 4.45–4.37 (1H, dd, cis-CH_2_), 6.44–6.87 (2H, m, Ar-H), 7.47–7.51 (1H, t, −CH=), 7.87–8.04 (2H, m, Ar-H). ^13^C NMR (CDCl_3_, δ, ppm): 142.8, 134.6, 132.8, 114.2, 113.8, 97.6, 80.4. LC–MS (ESI): m/z calcd. for C_10_H_10_O_3_^+^: 178.0708; found: 179.0703.

#### Vinyl ester of 4-fluorobenzoic acid (3d)

Yield: 83%; colorless liquid; Bp: 79–80°C / 50 mmHg; Rf = 0.50. FTIR (cm^−1^): 2926, 2861, 1713, 1643, 1384, 1253, 1089. ^1^H NMR (400 MHz, Pyridine-d_5_, δ, ppm): 5.00–5.06 (1H, dd, trans–CH_2_), 5.13–5.15 (1H, dd, cis–CH_2_), 7.12 (2H, m, Ar-H), 7.21 (1H, dd, Ar-H), 7.38 (1H, t, vinyl CH), 8.67 (1H, dd, Ar-H). ^13^C NMR (Pyridine-d_5_, δ, ppm): 155.8, 151.4, 133.7, 129.9, 123.6, 112.2, 97.7.

#### Vinyl ester of 4-tert-butylbenzoic acid (3e)

Yield: 65%; light yellow oily liquid; Bp: 75–77°C; Rf = 0.65. FTIR (cm^−1^): 3110, 2857, 1953, 1739, 1648, 1600, 1529, 1412, 1354, 1298, 1270, 1134, 1103. ^1^H NMR (400 MHz, CD_3_OD, δ, ppm): 1.43 (9H, s, t-Bu), 4.65 (1H), 5.03 (1H, dd, J = 7.8, 2.3 Hz), 5.13 (1H, dd, J = 14.8, 2.3 Hz), 7.32 (2H, ddd, J = 8.5, 1.7, 0.5 Hz), 7.44 (1H, dd, J = 14.8, 7.8 Hz), 7.90 (2H, ddd, J = 8.5, 1.7, 0.5 Hz). ^13^C NMR (CD_3_OD, δ, ppm): 31.3, 36.3, 95.7, 125.1, 129.4–129.7, 133.1, 142.8, 141.5, 171.0.

#### Vinyl ester of 4-chlorobenzoic acid (3f)

Yield: 85%; light yellow liquid; Bp: 63–64°C / 50 mmHg; Rf = 0.31. ^1^H NMR (400 MHz, CDCl_3_, δ, ppm): 8.09–7.98 (2H, m), 7.50 (1H, dd, J = 13.9, 6.2 Hz), 7.48–7.41 (2H, m), 5.08 (1H, dd, J = 13.9, 1.8 Hz), 4.73 (1H, dd, J = 6.2, 1.8 Hz). ^13^C NMR (CDCl_3_, δ, ppm): 162.8, 141.4, 140.2, 131.4, 129.0, 127.6, 98.6.

#### Divinyl ester of ortho-phthalic acid (3g)

Yield: 88%; white solid. ^1^H NMR (400 MHz, CDCl_3_, δ, ppm): 5.03 (2H, dd, vinyl CH_2_, cis), 5.13 (2H, dd, vinyl CH_2_, trans), 7.44 (2H, dd, vinyl CH), 7.68 (1H, td, Ar-H), 8.15 (2H, dt, Ar-H), 8.61 (1H, td, Ar-H). ^13^C NMR (CDCl_3_, δ, ppm): 164.1, 140.2, 133.8, 132.4, 129.3, 128.5, 98.2. FTIR (cm^−1^): 1089 (C–O–C), 1437 (C–H bend), 1667 (CH=CH_2_), 1714 (C=O), 2864 (CH_2_), 2927 (=CH).

#### Divinyl ester of terephthalic acid (3h)

Yield: 91%; white solid. ^1^H NMR (400 MHz, CDCl_3_, δ, ppm): 5.03 (2H, dd, vinyl CH_2_, cis), 5.13 (2H, dd, vinyl CH_2_, trans), 7.44 (2H, dd, vinyl CH), 7.77 (4H, ddd, Ar-H). ^13^C NMR (400 MHz, CD_3_OD, δ, ppm): 97.3 (2C, CH_2_), 129.5 (2C, Ar C-1,4), 129.6 (4C, Ar C-2,3,5,6), 141.3 (2C, vinyl CH), 163.5 (2C, −COO−).

## 3. Results and discussion

### 3.1. Morphological and structural characteristics of the synthesized catalytic systems

Modern analytical techniques, such as XRD, Raman spectroscopy, scanning electron microscopy (SEM), and energy-dispersive X-ray spectroscopy (EDX) were used to assess the structural and morphological properties of the synthesized Zn/SiOC, ZnO/SiOC, and Ni/SiOC catalytic systems.

**Figure 1:**
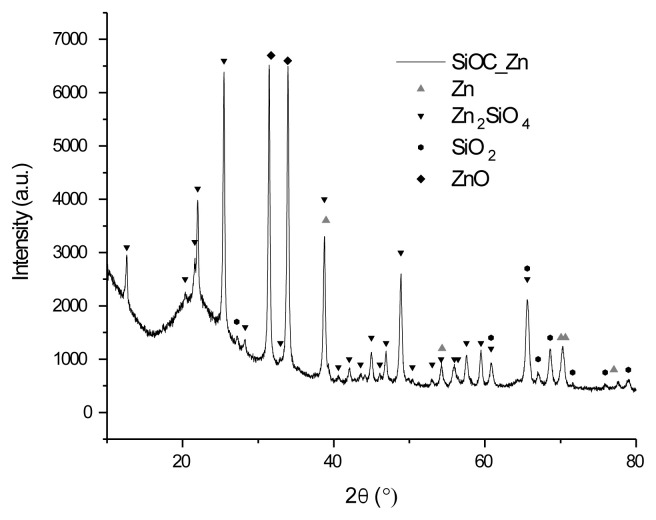
XRD spectrum of pyrolyzed Zn/SiOC.

**Figure 2:**
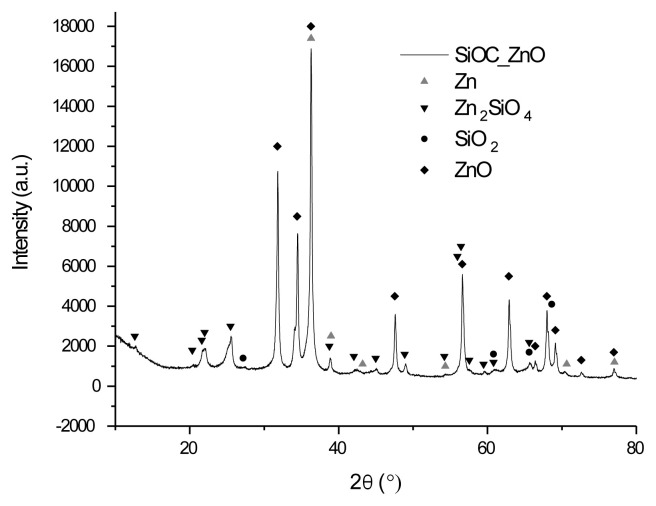
XRD spectrum of pyrolyzed ZnO/SiOC.

**Figure 3:**
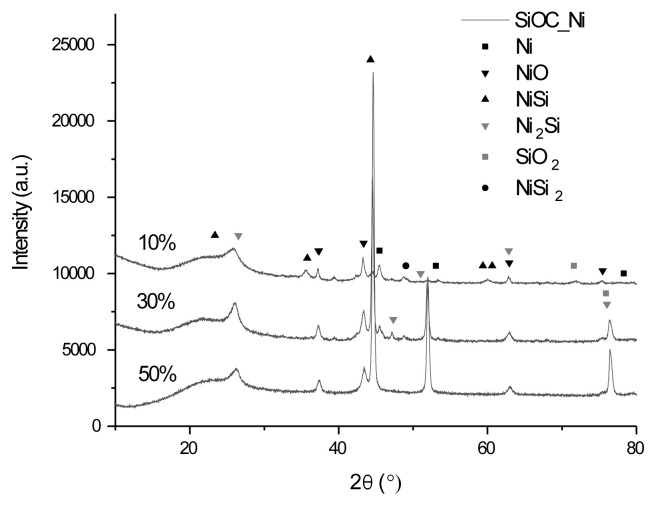
XRD spectra of pyrolyzed Ni/SiOC with different concentrations of Ni.

The XRD spectra of the Zn/SiOC, ZnO/SiOC, and Ni/SiOC samples display characteristic peaks that correspond to metallic or metal oxide phases (Figures 1–3). The presence of metallic zinc phases is indicated by the peaks both in the Zn/SiOC and ZnO/SiOC spectra at 2θ ≈ 38.9, 54.3, 70.1 and 70.6 (Figure 1), and according to peak intensities, in the ZnO doped composite we observe a low presence of metal component due to peculiarities of synthesis for all concentrations of dopants. The prevailing phase is the zinc silicate (willemite, Zn_2_SiO_4_), which can form in the resulting system during heat treatment and is also catalytically active. The analysis also revealed the presence of zinc oxide and silicon oxide (cristobalite) phases in the Zn/SiOC and ZnO/SiOC composites. The peaks confirmed the formation of wurtzite structure of ZnO are observed in ZnO/SiOCs XRD spectrum, as well as ZnO formed as additional phase in Zn/SiOC during heat treatment.

The peaks for Ni/SiOC at roughly 2θ ≈ 45.5, 53.0, and 78.3 show the presence of metallic nickel (Figure 3). Additionally, the XRD spectra of the Ni/SiOC composite contain peaks that correspond to additional phases inherent in its synthesis, such as oxides and silicides of the main cations. Unlike the previous samples, the volume of these additional phases changes more clearly depending on the amount of Ni. In each case, a broad peak in the 20–30 2θ range, characteristic of the SiOC matrix, suggests the presence of an amorphous structure.

The Raman spectra provided additional confirmation of the XRD data (Figures S1–S3). The signals found at roughly 1350 cm^−1^ (D-band) and 1580 cm^−1^ (G-band) indicate amorphous and graphitic carbon structures. The D/G intensity ratio shows some structural disorder, which may affect the catalytic activity. The absence of distinct peaks corresponding to crystalline oxides suggests that the metal/oxide phases are either widely distributed or exist in nanoscale dimensions.

**Figure 4:**
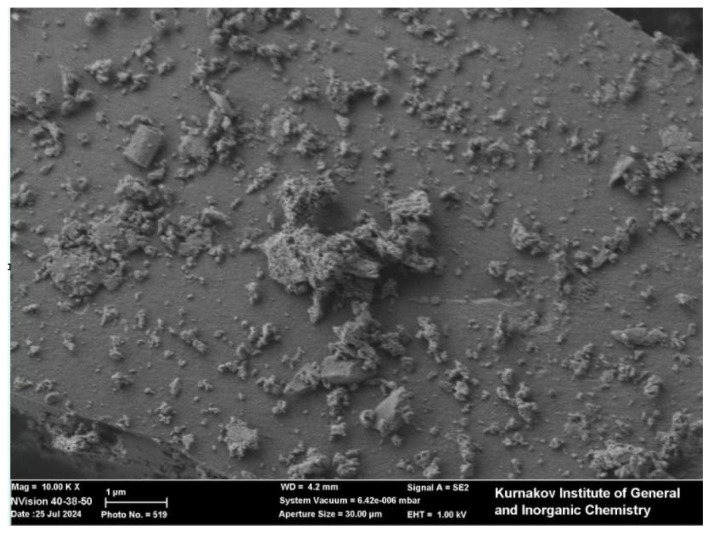
SEM image of ZnO/SiOC.

**Figure 5:**
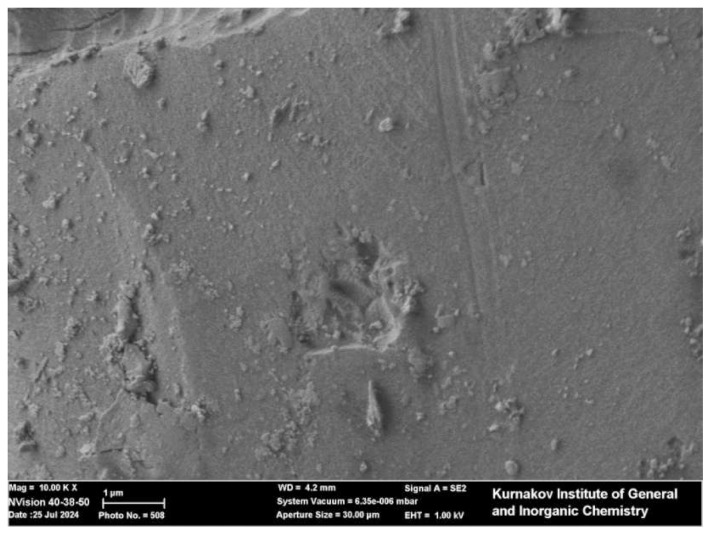
SEM image of Zn/SiOC.

According to the SEM images, the catalytic samples morphology is relatively uniform, ultrafine, and structurally porous in all cases. Tiny particle clusters were observed in the Zn/SiOC and ZnO/SiOC samples (Figures 4 and 5). These porous architectures promote process efficiency by increasing the availability of catalytically active sites.

**Figure 6:**
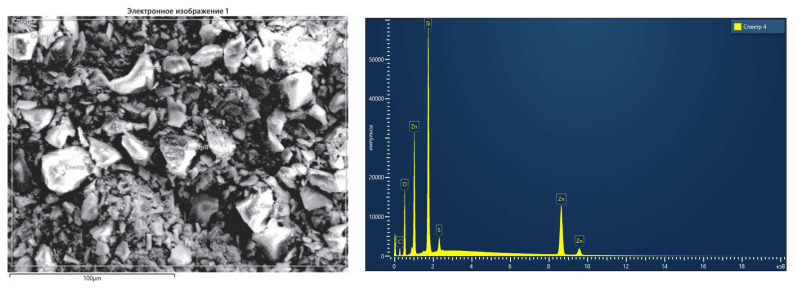
EDX analysis of Zn/SiOC.

The samples contained Zn, Ni, Si, O, and C, which were evenly distributed according to the EDX analysis (Figure 6). The successful incorporation of the catalytic components into the matrix was demonstrated by the atomic percentages of the elements. The relatively high oxygen content observed in the EDX data confirms the formation of the oxide phase in the ZnO/SiOC sample. The nickel in the Ni/SiOC system was found to be evenly distributed and present in a quantity that matched the intended low loading.

The conducted analyses confirm that the Zn/SiOC, ZnO/SiOC, and Ni/SiOC catalytic systems possess highly dispersed and porous structures, as well as efficient incorporation of the active phase elements. This, in turn, demonstrates the high potential of effective application of these catalytic systems in the vinylation reactions of aromatic carboxylic acids with acetylene.

### 3.2. Vinylation reactions of aromatic carboxylic acids in the presence of Zn/SiOC, ZnO/SiOC, and Ni/SiOC catalytic systems

Over the past few years, metal nanoparticles dispersed in silicon oxycarbide (SiOC) matrices have gained significant attention for use in organic synthesis due to their high thermal stability and large specific surface area, reaction selectivity, and unique catalytic properties. Nanostructured catalytic systems based on SiOC with embedded metals, such as Pd, Pt, and Au are widely used in hydrogenation, redox reactions, and C–C bond-forming reactions, such as Suzuki, Heck, and Sonogashira couplings [18]. Zn/SiOC and Ni/SiOC nanostructured systems have also been shown to play important roles in various organic transformations. These catalytic systems are used for the hydrogenation and dehydrogenation of various unsaturated hydrocarbons, including alkenes, alkynes, and aromatic hydrocarbons, as well as in selective oxidation, alkylation, and acylation via the Friedel–Crafts method, carbon–carbon bond formation (Suzuki, Negishi, and Kumada reactions), and cyclization reactions, such as Diels–Alder and alkene metathesis reactions [19–21].

In this work, vinyl esters were synthesized for the first time via the vinylation reactions of various substituted 1 with 2 under heterogeneous catalytic conditions using Zn/SiOC, ZnO/SiOC, and Ni/SiOC catalytic systems. The yield of the synthesized vinyl esters was investigated with respect to the nature of the starting materials, temperature, reaction duration, and nature of solvent and catalysts. Silicon oxycarbide-based catalytic systems (Zn/SiOC, ZnO/SiOC, and Ni/SiOC) were applied after being impregnated onto a silicon carbide (SiC) support.

From the substrates 1: benzoic (1a), 4-methylbenzoic (1b), 4-methoxybenzoic (1c), 4-fluorobenzoic (1d), 4-tert butylbenzoic (1e), 4-chlorobenzoic (1f), ortho phthalic (1g), and terephthalic (1h) acids—the corresponding vinyl esters were synthesized under heterogeneous catalytic conditions with 2. These include the vinyl ester of acids: benzoic (3a), of 4-methylbenzoic (3b), of 4-methoxybenzoic (3c), of 4-fluorobenzoic (3d), of 4-tert butylbenzoic (3e), of 4-chlorobenzoic (3f), divinyl ester of ortho phthalic (3g), and divinyl ester of terephthalic (3h). The general reaction scheme is presented in Scheme.

The reaction proceeds as follows. Reactants 2 and 1 are adsorbed onto the surface of the catalytic system. Highly efficient adsorption is ensured by the unique properties and high surface area of SiOC. The molecules of 2 and carboxylic acid are activated by the catalytic system. The metal active centers (Zn, ZnO, or Ni) in the SiOC matrix coordinate with the carboxyl group and the π-electrons of 2, thereby forming an activated complex. This activated species of 2 undergoes nucleophilic attack on the carboxyl group of 1, leading to the formation of the corresponding vinyl ester, which subsequently desorbs from the catalyst surface, regenerating the active sites for further catalytic cycles.

The structures of the synthesized compounds 3a–h were confirmed by FTIR, ^1^H NMR, ^13^C NMR, and chromatographic-mass spectral analyses (Figures S4–S19). Analysis of the ^1^H NMR spectra of the synthesized 3a–h revealed two doublet signals characteristic of the −CH_2_ group in the vinyl moiety in the range of 3.76–4.99 ppm, and chemical shift signal for the −CH proton of the vinyl group in the range of 7.14–7.67 ppm. Additionally, in the FTIR spectrum, absorption bands characteristic for the vinyl group (−CH=CH_2_) were observed in the 1645–1690 cm^−1^ region [22].

For the vinylation reaction of various substituted 1a–h with 2, silicon oxycarbide-based catalytic systems loaded with 50% Zn, ZnO, or Ni (Zn/SiOC-50, ZnO/SiOC-50, Ni/SiOC-50) were used in DMF solution at 150 C for 12 h. The starting acids 1a–h and 2 were used in 1:2 molar ratio, while the catalytic system loading was 10 mol% relative to the 1a–h. The results obtained are presented in Table 1.

In the vinylation of 1a–h with 2 using Zn/SiOC, ZnO/SiOC, and Ni/SiOC catalytic systems, the synthesis of 3a–h is influenced by various factors, including the acidity of the carboxylic acid and the electronic and steric nature of ring substituents. It was observed that the yield of 3a–h increased in the order of catalytic systems: Ni/SiOC-50 < ZnO/SiOC-50 < Zn/SiOC-50. The Zn/SiOC-50 system demonstrates the highest catalytic activity, which can be attributed to the ability of the positively charged zinc ions to coordinate with the π-bond of the alkyne in 2, thereby facilitating the formation of the active complex. Furthermore, increasing the acidity of the carboxylic acid results in a higher vinyl ester yield. Stronger acids facilitate the vinylation process by donating protons more easily, which accelerates the formation of reactive intermediates. Vinyl ester yields were higher for carboxylic acids with electron-withdrawing substituents (−Cl, −F) compared to those with electron-donating groups (−CH_3_). Additionally, the methoxy group donates electrons and reduces the acidity of the carboxylic acid through a strong positive mesomeric (+M) effect, resulting in a lower vinyl ester yield. The steric hindrance of the tert-butyl group also has negatively affected the product yield. When the vinylation reaction was carried out at the Zn/SiOC-50 catalytic system, the corresponding yields were as follows: *3*a-80%, *3b*-77%, *3c*-70%, *3d*-83%, *3e*-65%, *3f*-85%, *3g*-88%, and *3h*-91%.

The mass fraction of metal in silicon oxycarbide catalytic systems such as Ni/SiOC, ZnO/SiOC, and Zn/SiOC significantly affects the yield of 3a–h in the vinylation reactions of 1a–h with 2. The metal content influences the number of active sites available for the reaction, the distribution of the metals distribution within the SiOC matrix pores, and overall catalytic efficiency. The effect of the zinc content (10, 30, 50, and 70 mass%) in the Zn/SiOC system on vinyl ester yield is summarized in Table 2.

The results demonstrate that the Zn/SiOC-10 catalytic system exhibited the lowest activity. Significant improvement in yield was observed with the Zn/SiOC-30 system, while the Zn/SiOC-50 system produced the highest vinyl ester yields due to an optimal number of active sites and uniform metal dispersion. However, excessive zinc loading in the Zn/SiOC-70 system led to agglomeration and decreased accessibility to active sites, reducing catalytic activity and product yield. Therefore, all subsequent experiments were carried out using the Zn/SiOC-50 catalytic system.

The amount of catalyst used in the vinylation reaction of carboxylic acids with 2 significantly influences the yield of 3a–h. The effect of varying catalyst loadings (5, 10, and 15 mol%) of the Zn/SiOC-50 catalytic system on the synthesis of 3a–h was systematically investigated.

**Figure 7:**
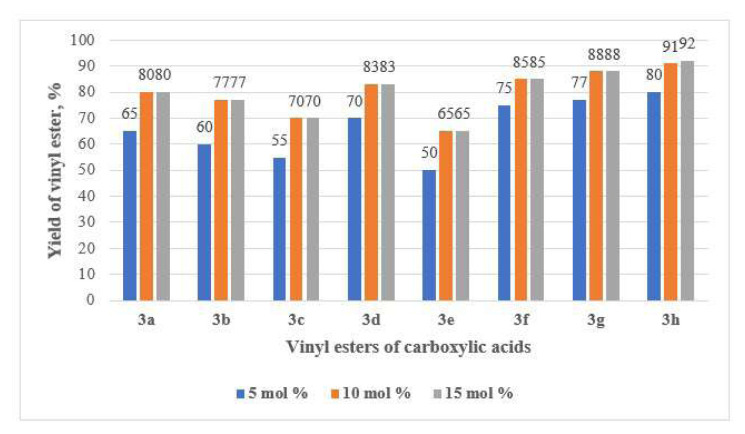
Effect of catalyst amount on vinyl ester yield. Catalytic system: Zn/SiOC-50; temperature: 150 C; reaction duration: 12 h; solvent: DMF.

As shown in Figure 7, the addition of 10°mol% Zn/SiOC-50 as the catalytic system yielded the highest product yield in the synthesis of vinyl esters in all experiments. As observed, increasing the catalyst loading from 5 to 10°mol% led to a notable rise in yields: from 65% to 80% for 3a, 60% to 77% for 3b, 55% to 70% for 3c, 70% to 83% for 3d, 50% to 65% for 3e, 75% to 85% for 3f, 77% to 88% for 3g, and 80% to 91% for 3h. However, increasing the catalyst loading to 15°mol% did not improve the yield any further, indicating that the optimal catalytic activity was achieved at 10°mol%.

The effect of temperature on the vinyl ester synthesis was also examined for the vinylation of 1a–h with 2 using the Zn/SiOC-50 catalytic system (Table 3).

The reaction was carried out at temperature ranging from 50 to 200°C. At 50 and 100°C, the yields were low because the reactant molecules and catalytic sites lacked sufficient energy for effective collisions. Above 100°C and up to 150°C, the yields of the product significantly improved: for 3a from 40% to 80%, 3b from 35% to 77%, 3c from 32% to 70%, 3d from 45% to 83%, 3e from 30% to 65%, 3f from 50% to 85%, 3g from 45% to 88%, and 3h from 42% to 91%. Overall, increasing the reaction temperature has enhanced both the reaction rate and the yield of vinyl esters 3a–h. However, raising the temperature to 200°C resulted in the formation of side products and potential catalyst deactivation, which ultimately decreased the vinyl ester yields.

The effect of reaction time on the vinylation of 1 with 2 was also investigated. To evaluate its influence on the yields of vinyl esters 3a–h, the reactions were conducted over various time intervals ranging from 6 to 15 h.

**Figure 8:**
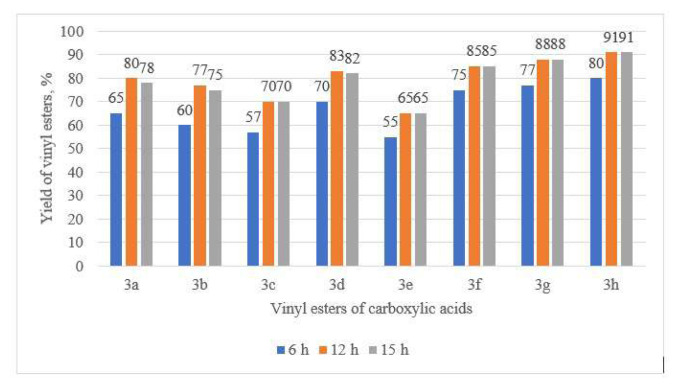
Influence of reaction duration on vinyl ester yield. Temperature: 150 C; solvent: DMF; catalyst: Zn/SiOC-50 at 10 mol%.

As shown in Figure 8, when the reaction was carried out for 6 h, it did not reach completion, resulting in low yield of 3a–h. When the reaction time was increased to 12 h, vinylation of the carboxylic acid proceeded to completion, and the maximum yields of 3a–h were achieved: *3a*–80%, *3b*–77%, *3c*–70%, *3d*–83%, *3e*–65%, *3f*–85%, *3g*–88%, and *3h*–91%. Further increase of reaction did not result in any significant improvement in product yield.

In the synthesis of 3a–h via the vinylation reaction of carboxylic acids with 2 using the Zn/SiOC-50 catalyst system, the nature of the solvent plays a crucial role. Solvents influence the solubility of reactants, the stability of intermediates, and the overall reaction environment, thereby affecting reaction rate, yield, and selectivity. For the vinylation reaction of carboxylic acids with 2, polar protic solvents commonly used in organic synthesis such as DMF and DMSO were employed. These solvents are known for their high boiling points, ability to dissolve both organic and inorganic substances, and their capacity to stabilize reaction intermediates [23].

**Figure 9:**
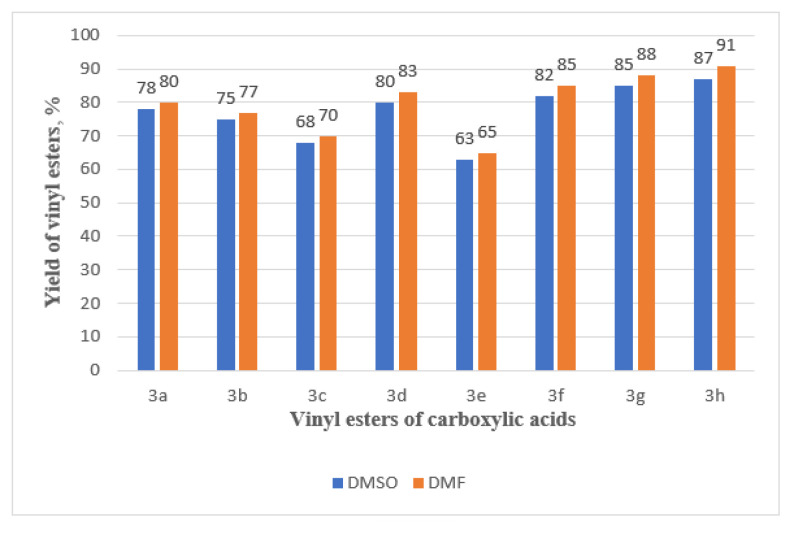
Effect of solvent nature on the yield of 3a–h. Temperature: 150 C; catalyst: Zn/SiOC-50 at 10 mol%; reaction duration: 12 h.

As shown in Figure 9, the results indicate that the 3a–h are obtained with marginally higher yields in DMF than in DMSO. This can be explained as follows: Although DMSO has a better solubilizing characteristic in comparison to DMF, it may not be as effective at maintaining the optimal concentration of reactants and intermediates in this particular reaction. DMF, with a dielectric constant of approximately 36.7, stabilizes charged transition states and intermediates efficiently, thereby increasing the heterogeneous catalytic vinylation reaction. Moreover, a larger dielectric constant of DMSO (approximately 46.7) could benefit some reactions; it could overstabilize some of the intermediates in this case, which in turn can negatively impact the reaction rate and yield. In the DMF, intermediate formation is preferred, and the coordination between the Zn/SiOC-50 catalytic system and the reactants is quicker, enhancing the reaction kinetics and yielding a higher overall product yield.

Subsequently, the reusability of Zn/SiOC, ZnO/SiOC, and Ni/SiOC catalytic systems was examined. Following each cycle of the reaction, the catalyst was recovered through filtration, cleaned with ethanol and DMF, and then dried at 80 C. Interestingly, the catalytic activity remained high, and the vinyl esters were synthesized with consistently high yields, even after five consecutive cycles. These results demonstrate the practical potential of these heterogeneous catalytic systems for economical and sustainable applications in organic synthesis due to their exceptional stability and recyclability. Compared to previously reported homogeneous or conventional catalytic systems, the Zn/SiOC-50 catalyst demonstrated significantly higher yields (up to 91%) under milder conditions. It also enabled catalyst recyclability of up to five cycles without significant loss of activity. These results highlight the practical advantages and novelty of the Zn/SiOC-50 catalyst in the vinylation of aromatic carboxylic acids. In comparison to previously reported homogeneous metal catalysts (e.g., Pd, Cu, Ni), the synthesized Zn/SiOC, ZnO/SiOC, and Ni/SiOC catalysts show high activity and stability in the vinylation of aromatic carboxylic acids, according to the studys results [24]. These catalysts are particularly promising substitutes in organic synthesis because of their environmental advantages, thermal stability, and reusability.

## 4. Conclusion

In this work, a novel process for the synthesis of vinyl esters from substituted 1 by 2 vinylation under heterogeneous catalytic conditions was investigated. The reactions were successfully carried out using silicon oxycarbide-based catalytic systems: Zn/SiOC, ZnO/SiOC and Ni/SiOC, and Zn/SiOC-50. A systematic investigation of the reaction parameters revealed that the reaction temperature, catalyst loading, reaction time, and nature of the solvent significantly influence the selectivity and yield of the process. The reactions were performed under the optimum conditions of 10 mol% of Zn/SiOC-50 at 150 C for a reaction time of 12 h in the of DMF solvent. Under these conditions 3a–h were obtained in high yields (80%–91%), depending on the substrate. Both low and high catalyst loadings negatively affected the yield, while 10 mol% provided the best balance between active sites and metal dispersion. Similarly, the reaction exhibited clear temperature dependence, with increasing yields up to 150 C, followed by side reactions and deactivation of the catalyst. The solvent effect was also critical: despite DMSOs higher dielectric constant, DMF outperformed DMSO in stabilizing charged intermediate species, enabling quicker reaction kinetics and, consequently, product yield. Overall, the result demonstrates the potential of employing Zn/SiOC-based catalytic systems for the effective vinylation reactions, offering an alternative, scalable method for the synthesis of vinyl esters from organic acids.

## Supporting information

Figure S1 FTIR spectrum of pyrolyzed Zn/SiOC.

Figure S2 FTIR spectrum of pyrolyzed ZnO/SiOC.

Figure S3 FTIR spectrum of pyrolyzed Ni/SiOC.

Figure S4 FTIR spectrum of vinyl ester of benzoic acid.

Figure S5 ^1^H NMR spectrum of vinyl ester of benzoic acid.

Figure S6 ^13^C NMR spectrum of vinyl ester of benzoic acid.

Figure S7 Chromato-mass spectrum of vinyl ester of benzoic acid.

Figure S8 ^1^H NMR spectrum of vinyl ester of 4-fluorobenzoic acid.

Figure S9 ^13^C NMR spectrum of vinyl ester of 4-fluorobenzoic acid.

Figure S10 FTIR spectrum of vinyl ester of 4-Methoxybenzoic acid.

Figure S11 ^1^H- NMR spectrum of vinyl ester of 4-methoxybenzoic acid.

Figure S12 ^13^C- NMR spectrum of vinyl ester of 4-methoxybenzoic acid.

Figure S13 FTIR spectrum of vinyl ester of 4-methylbenzoic acid.

Figure S14 ^1^H- NMR spectrum of vinyl ester of 4-methylbenzoic acid.

Figure S15 ^13^C NMR -spectrum of vinyl ester of 4-Methylbenzoic acid.

Figure S16 ^1^H-NMR spectrum of vinyl ester of 4-tert-butylbenzoic acid.

Figure S17 ^13^C NMR spectrum of vinyl ester of 4-tert-butylbenzoic acid.

Figure S18 FTIR spectrum of vinyl ester of 4-chlorobenzoic acid.

Figure S19 ^1^H-NMR spectrum of vinyl ester of 4-chlorobenzoic acid.

## Figures and Tables

**Scheme:**
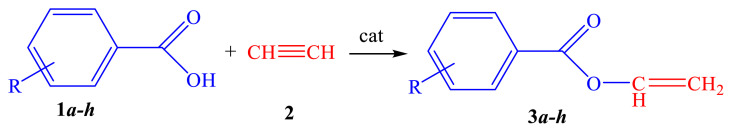
Vinylation reaction of aromatic carboxylic acids under heterogeneous catalytic conditions in the presence of Zn/SiOC, ZnO/SiOC, and Ni/SiOC.

**Table 1:** Influence of the nature of catalytic systems on the vinylation process.

№	Vinyl esters of carboxylic acids	Catalytic system / Yield of vinyl ester, %		

		Zn/SiOC-50	ZnO/SiOC-50	Ni/SiOC-50
1	***3a***	80	75	55
2	***3b***	77	70	50
3	***3c***	70	65	45
4	***3d***	83	80	65
5	***3e***	65	60	40
6	***3f***	85	83	70
7	***3g***	88	79	61
8	***3h***	91	85	74

**Table 2:** Dependence of vinyl ester yield on the zinc ion mass fraction in the Zn/SiOC catalytic system. Temperature:150 C; reaction time:12 h; solvent: DMF; catalyst loading:10 mol%.

№	Vinyl esters of carboxylic acids	Catalytic system / Yield of vinyl ester, %			

		Zn/SiOC-10	Zn/SiOC-30	Zn/SiOC-50	Zn/SiOC-70
1	***3a***	55	70	80	75
2	***3b***	52	68	77	72
3	***3c***	48	63	70	65
4	***3d***	58	73	83	78
5	***3e***	45	58	65	60
6	***3f***	60	75	85	80
7	***3g***	59	76	88	81
8	***3h***	65	78	91	85

**Table 3:** Effect of temperature on vinyl ester yield. Catalytic system: 10 mol% Zn/SiOC-50; reaction duration: 12 h; solvent: DMF.

№	Vinyl esters of carboxylic acids	Temperature, °C / Yield of vinyl ester,%			

		50	100	150	200
1	***3a***	40	60	80	75
2	***3b***	35	55	77	70
3	***3c***	32	53	70	65
4	***3d***	45	65	83	78
5	***3e***	30	50	65	60
6	***3f***	50	70	85	80
7	***3g***	45	65	88	80
8	***3h***	42	74	91	75
