# Supplementary figures and images for: Synthesis of vinyl esters of aromatic carboxylic acids in the presence of Zn/SiOC, ZnO/SiOC, and Ni/SiOC catalytic systems
Source: Turk J Chem. 2025 Aug 5;49(5):520–31. doi: 10.55730/1300-0527.3750 (PMC12604925; doi:10.55730/1300-0527.3750)

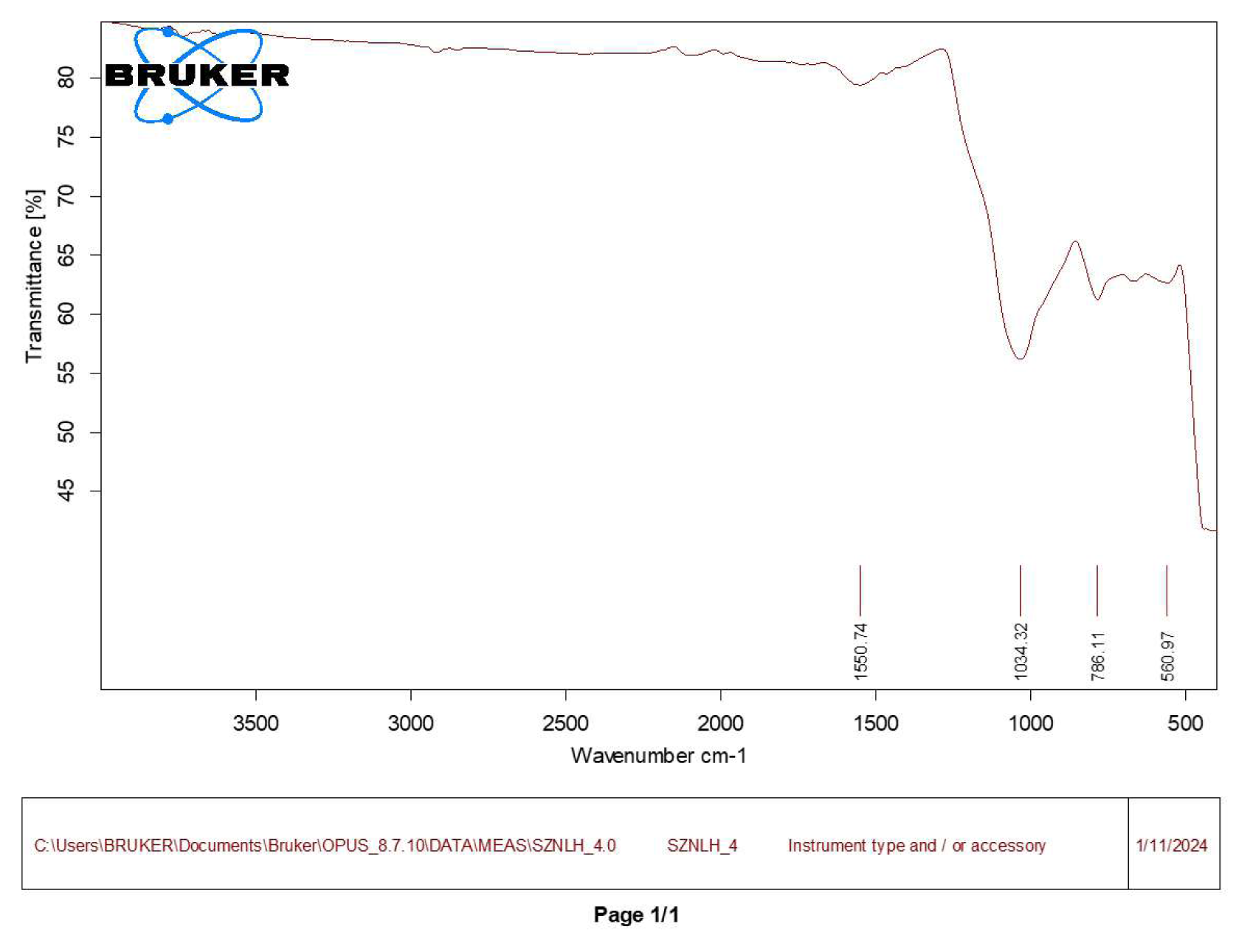

Supplement: Figure S1 — FTIR spectrum of pyrolyzed Zn/SiOC. [file tjc-49-05-520s1.tif]

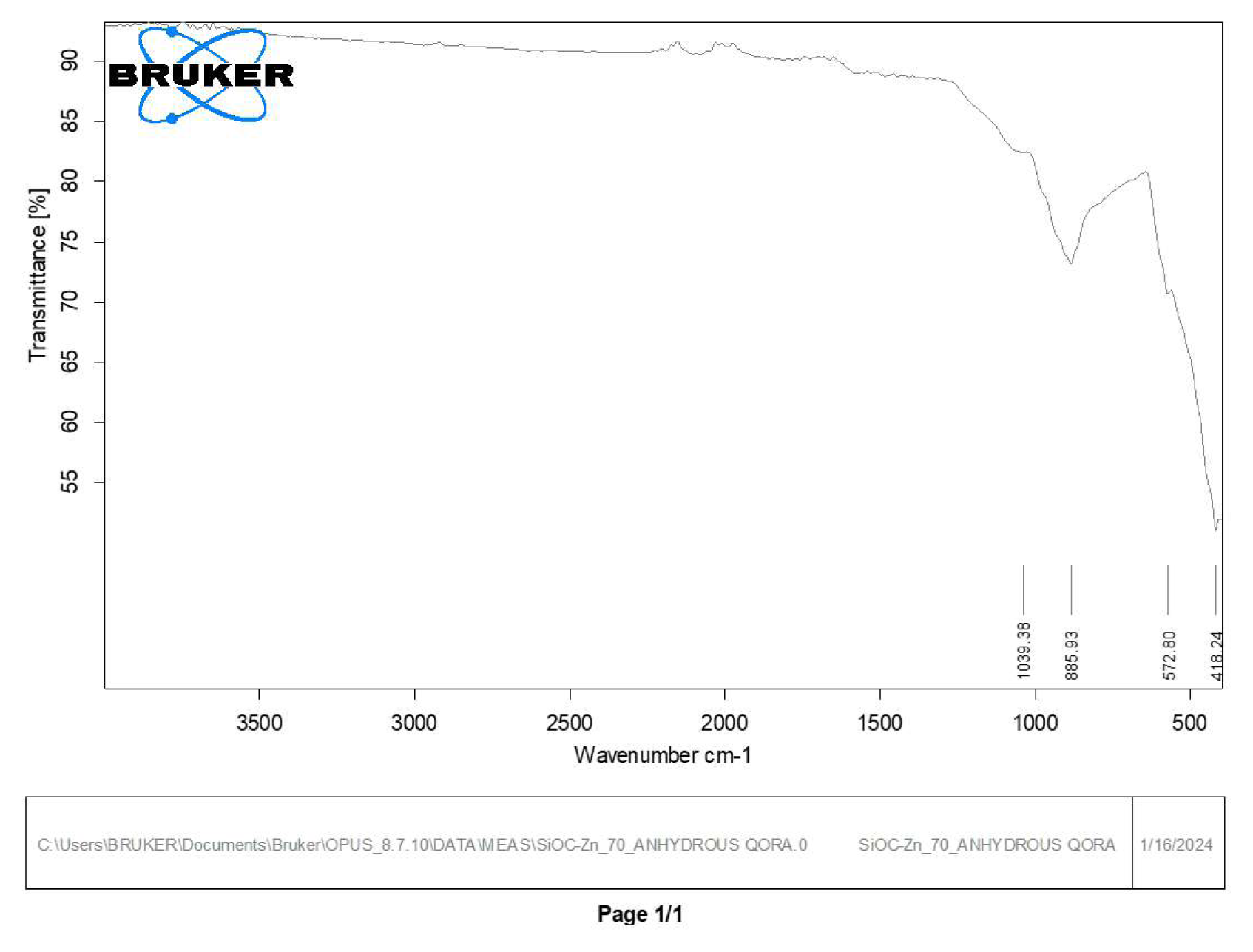

Supplement: Figure S2 — FTIR spectrum of pyrolyzed ZnO/SiOC. [file tjc-49-05-520s2.tif]

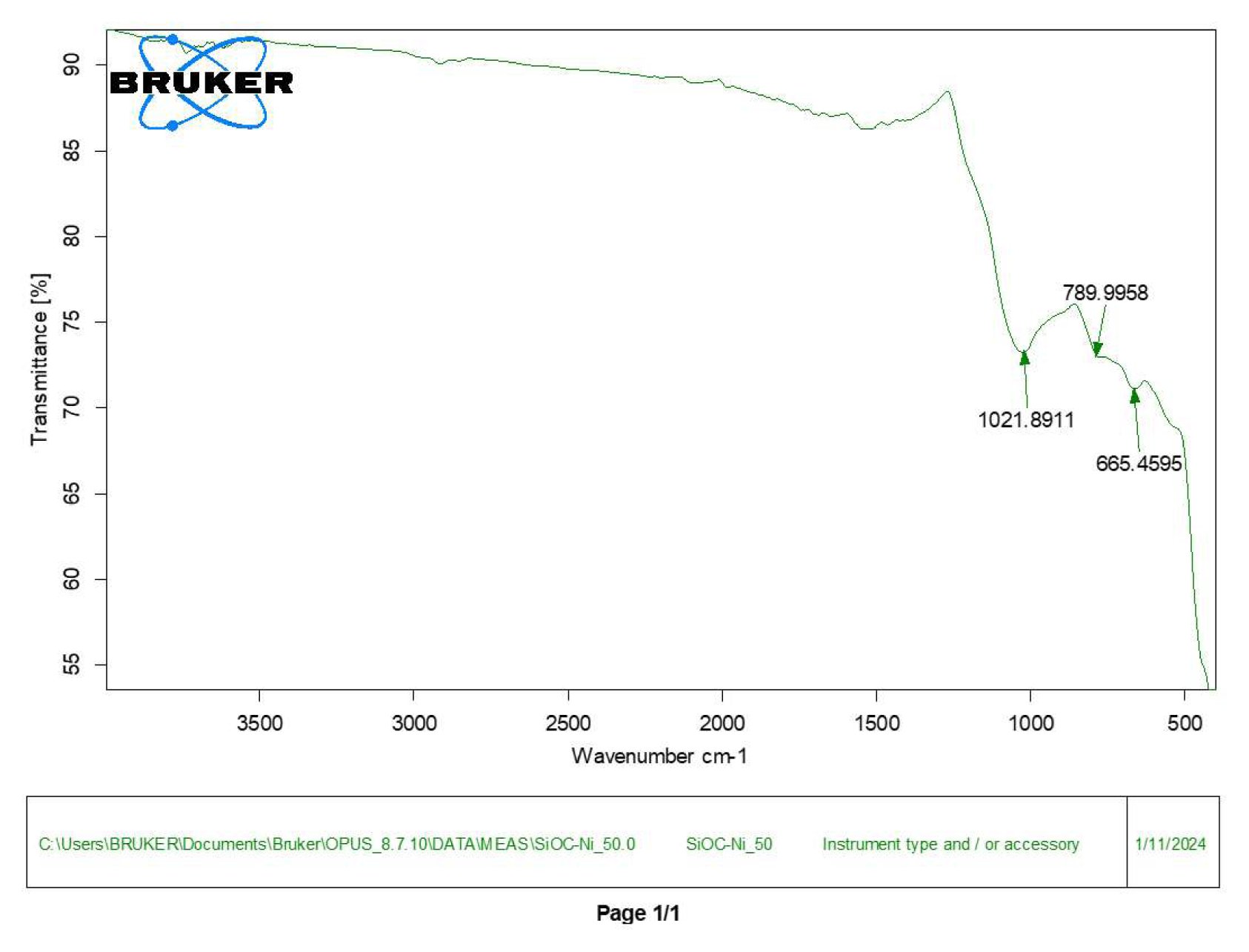

Supplement: Figure S3 — FTIR spectrum of pyrolyzed Ni/SiOC. [file tjc-49-05-520s3.tif]

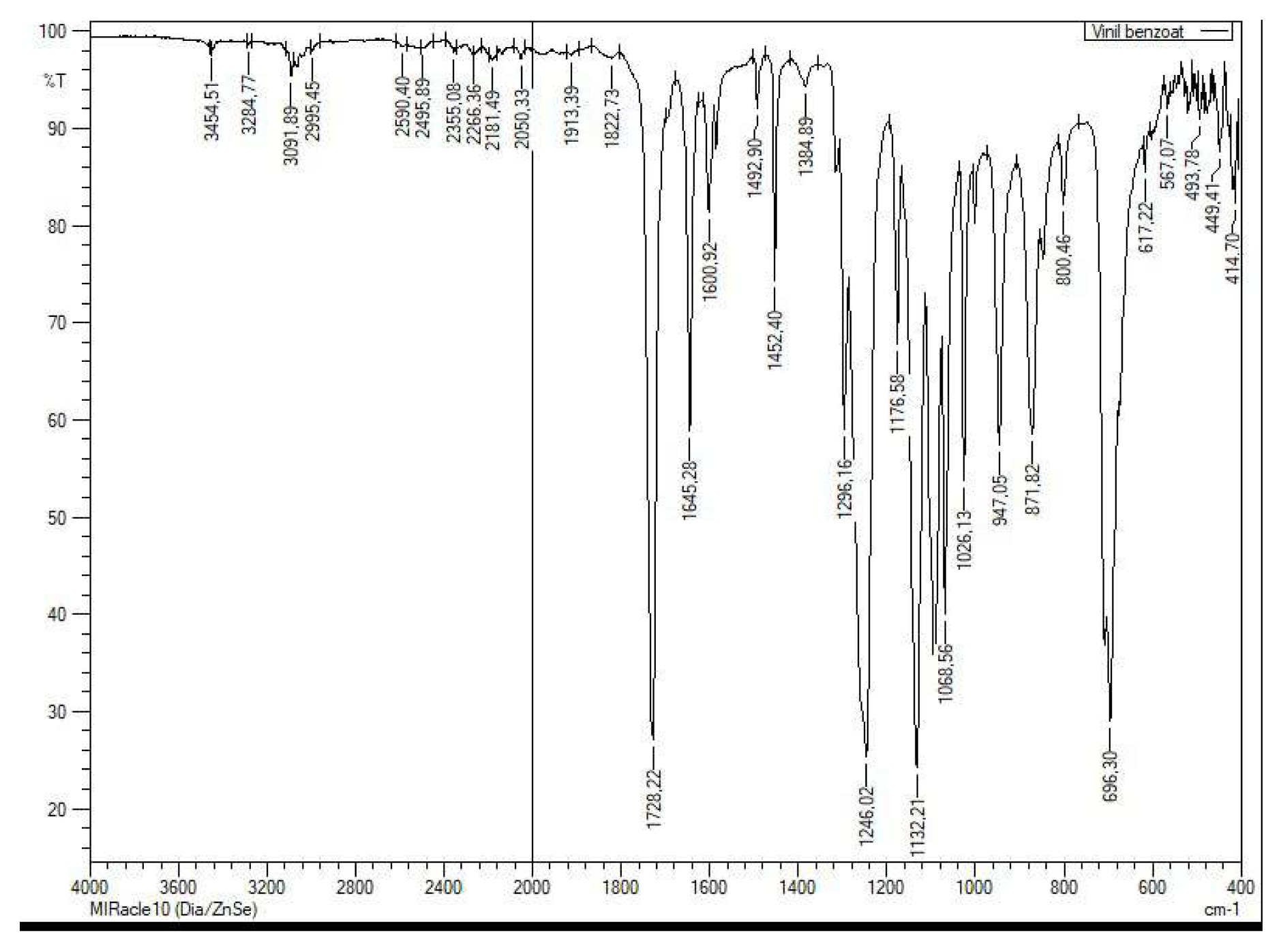

Supplement: Figure S4 — FTIR spectrum of vinyl ester of benzoic acid. [file tjc-49-05-520s4.tif]

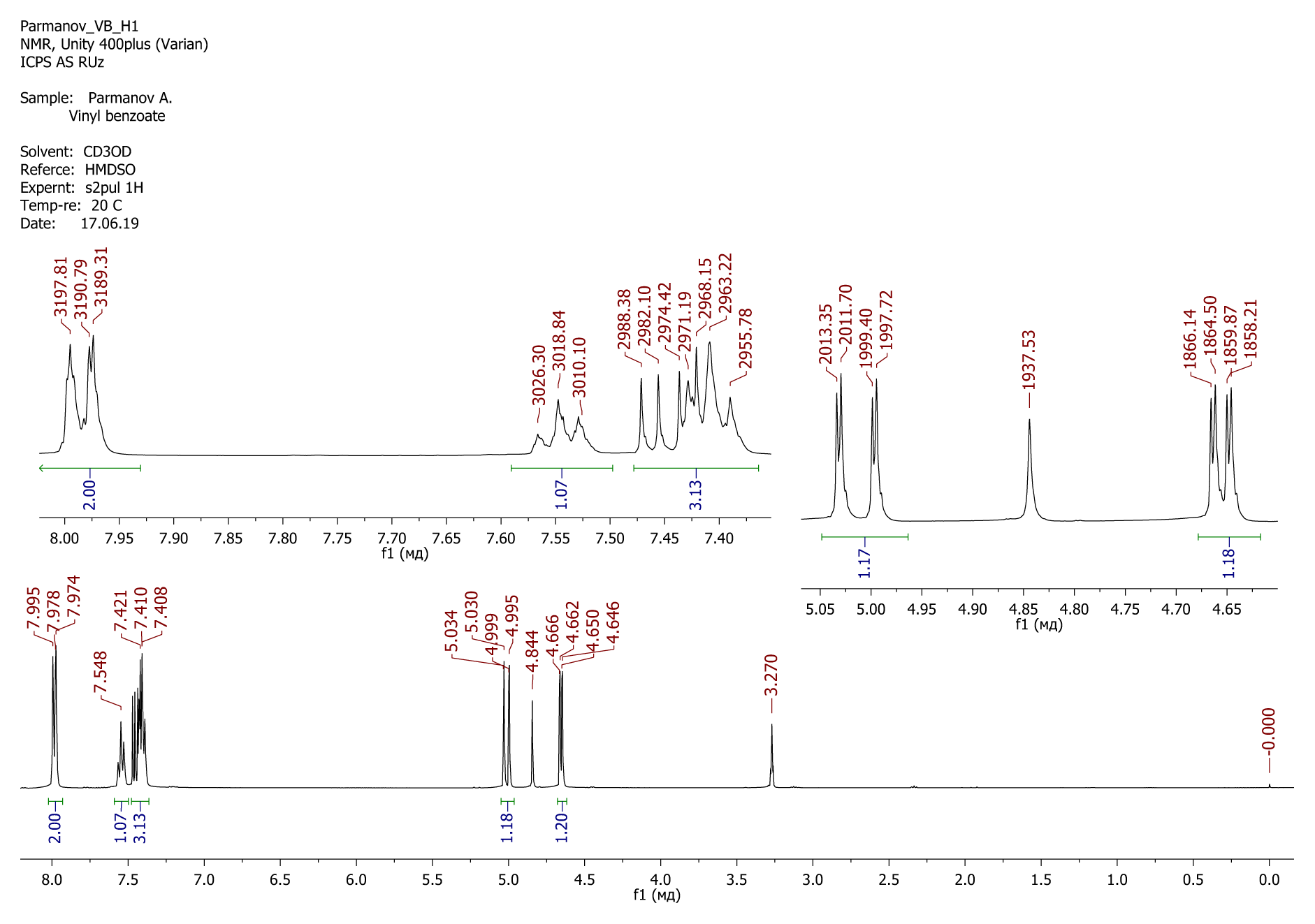

Supplement: Figure S5 — 1H NMR spectrum of vinyl ester of benzoic acid. [file tjc-49-05-520s5.tif]

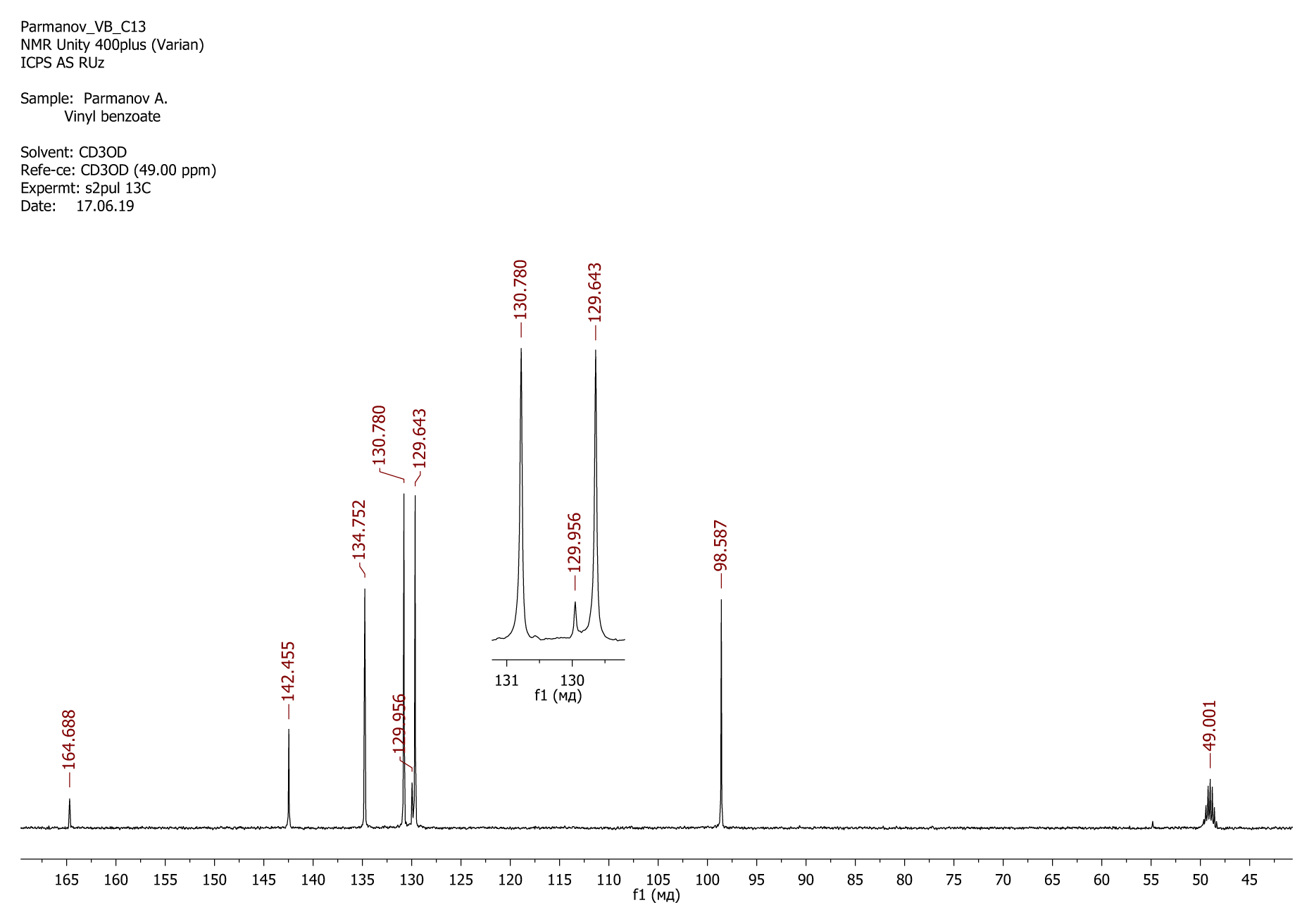

Supplement: Figure S6 — 13C NMR spectrum of vinyl ester of benzoic acid. [file tjc-49-05-520s6.tif]

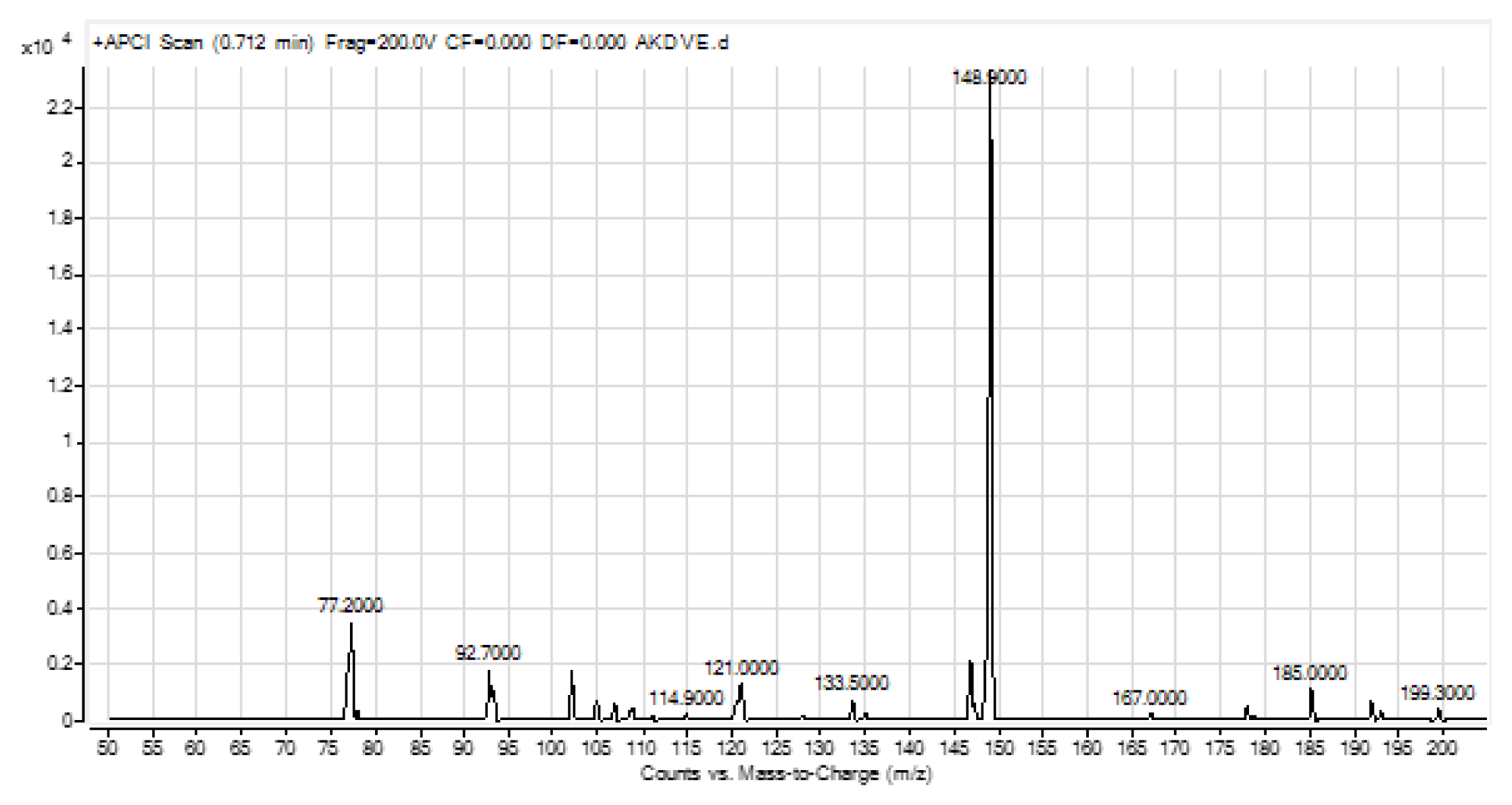

Supplement: Figure S7 — Chromato-mass spectrum of vinyl ester of benzoic acid. [file tjc-49-05-520s7.tif]

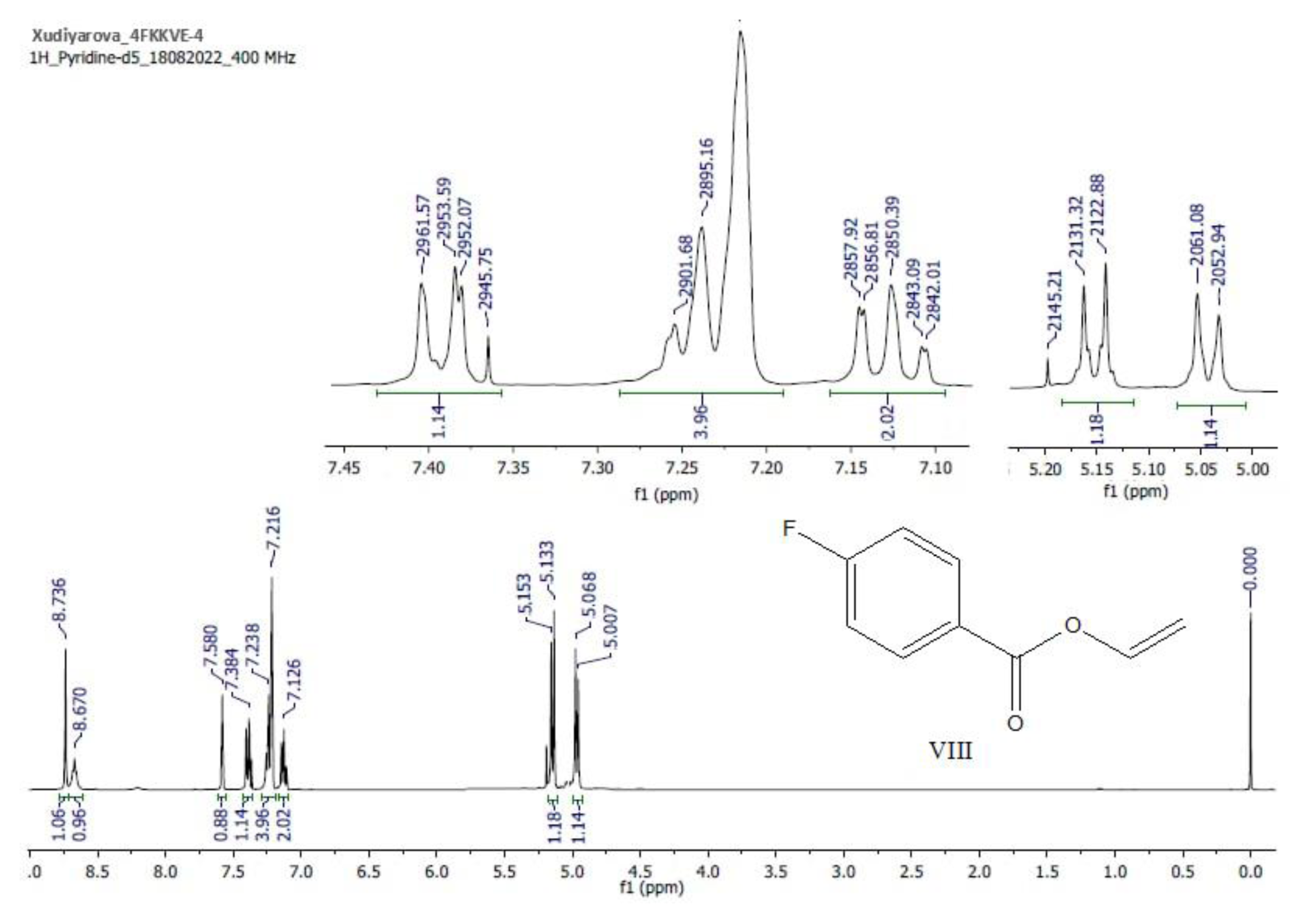

Supplement: Figure S8 — 1H NMR spectrum of vinyl ester of 4-fluorobenzoic acid. [file tjc-49-05-520s8.tif]

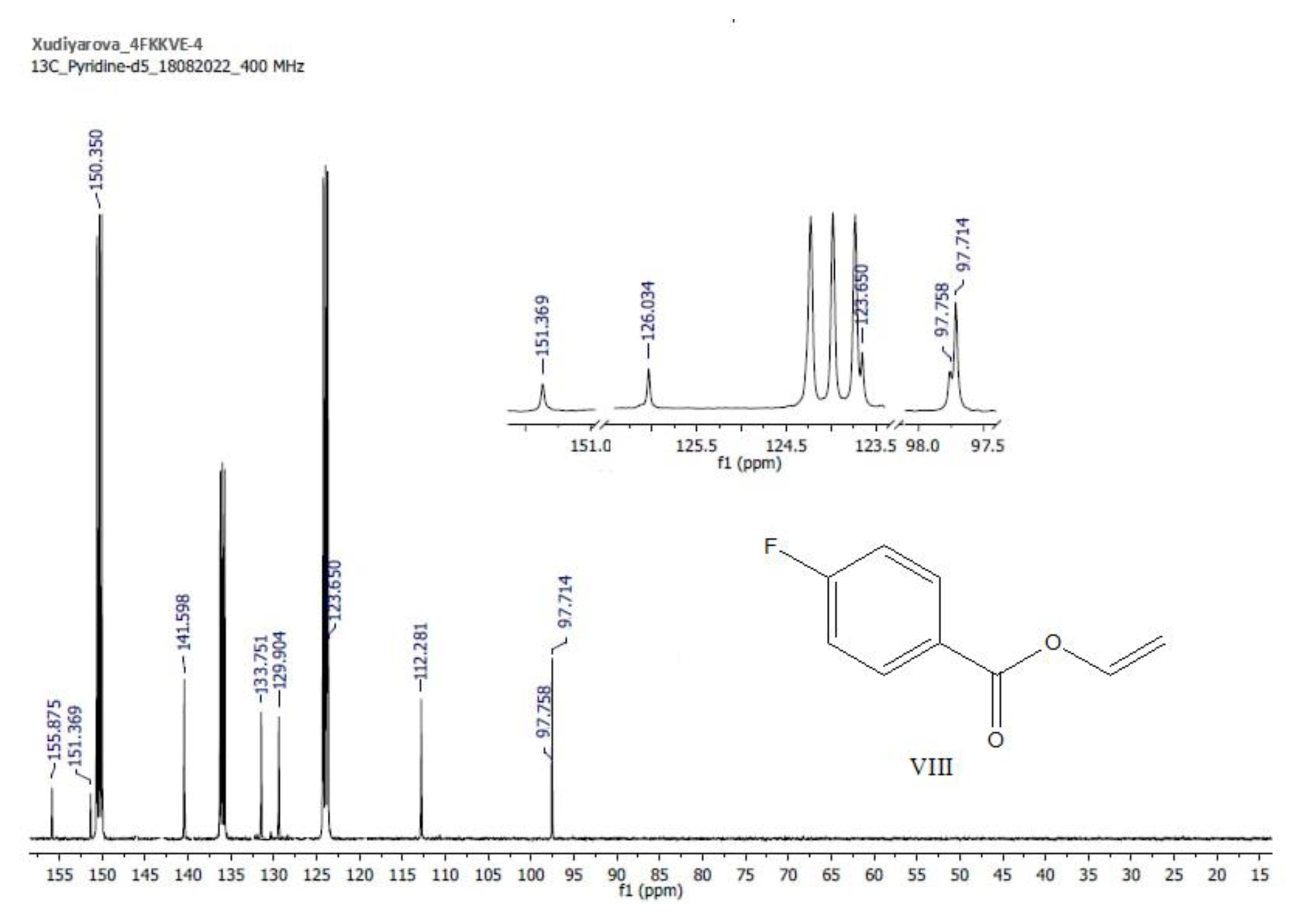

Supplement: Figure S9 — 13C NMR spectrum of vinyl ester of 4-fluorobenzoic acid. [file tjc-49-05-520s9.tif]

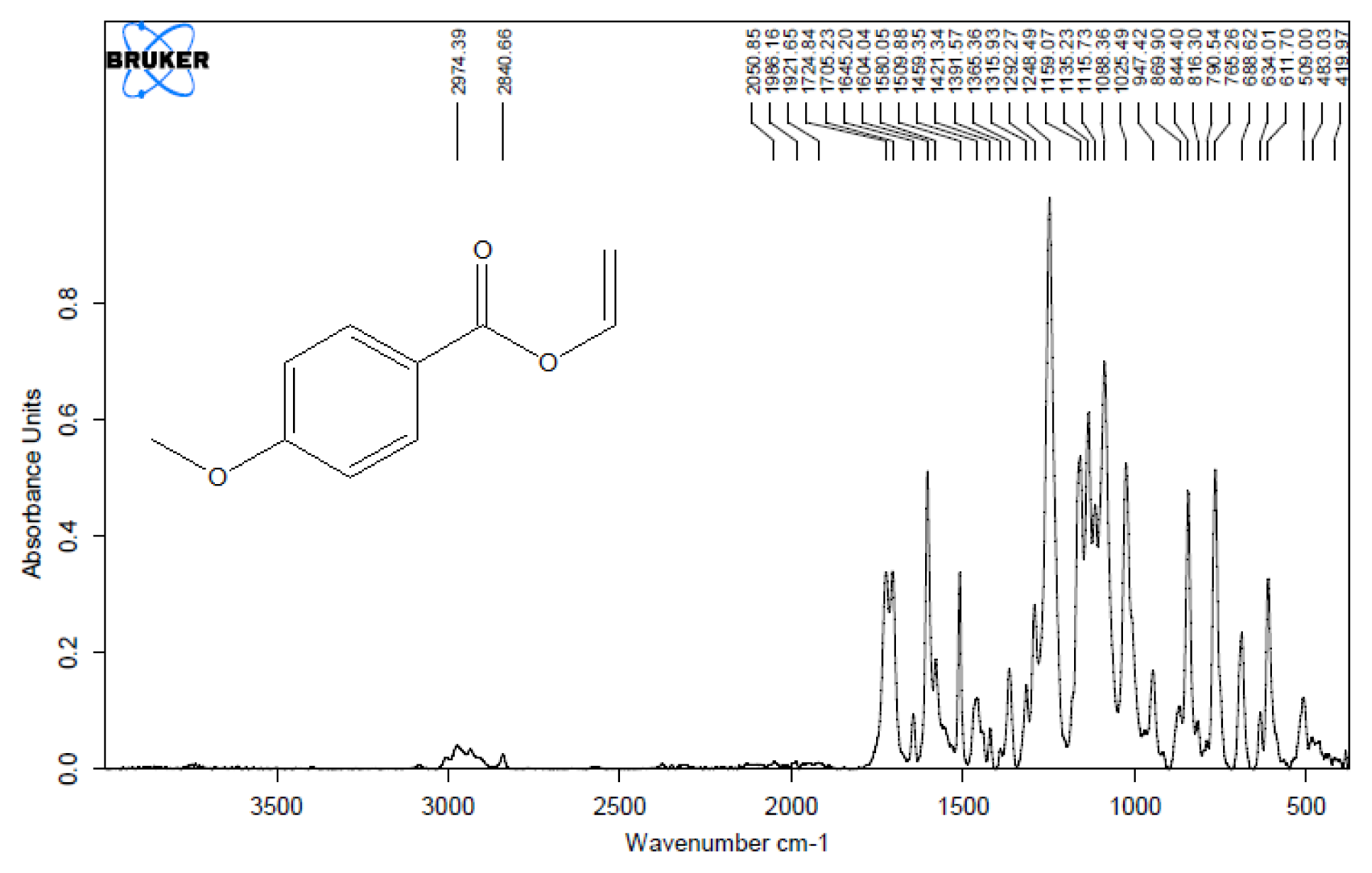

Supplement: Figure S10 — FTIR spectrum of vinyl ester of 4-Methoxybenzoic acid. [file tjc-49-05-520s10.tif]

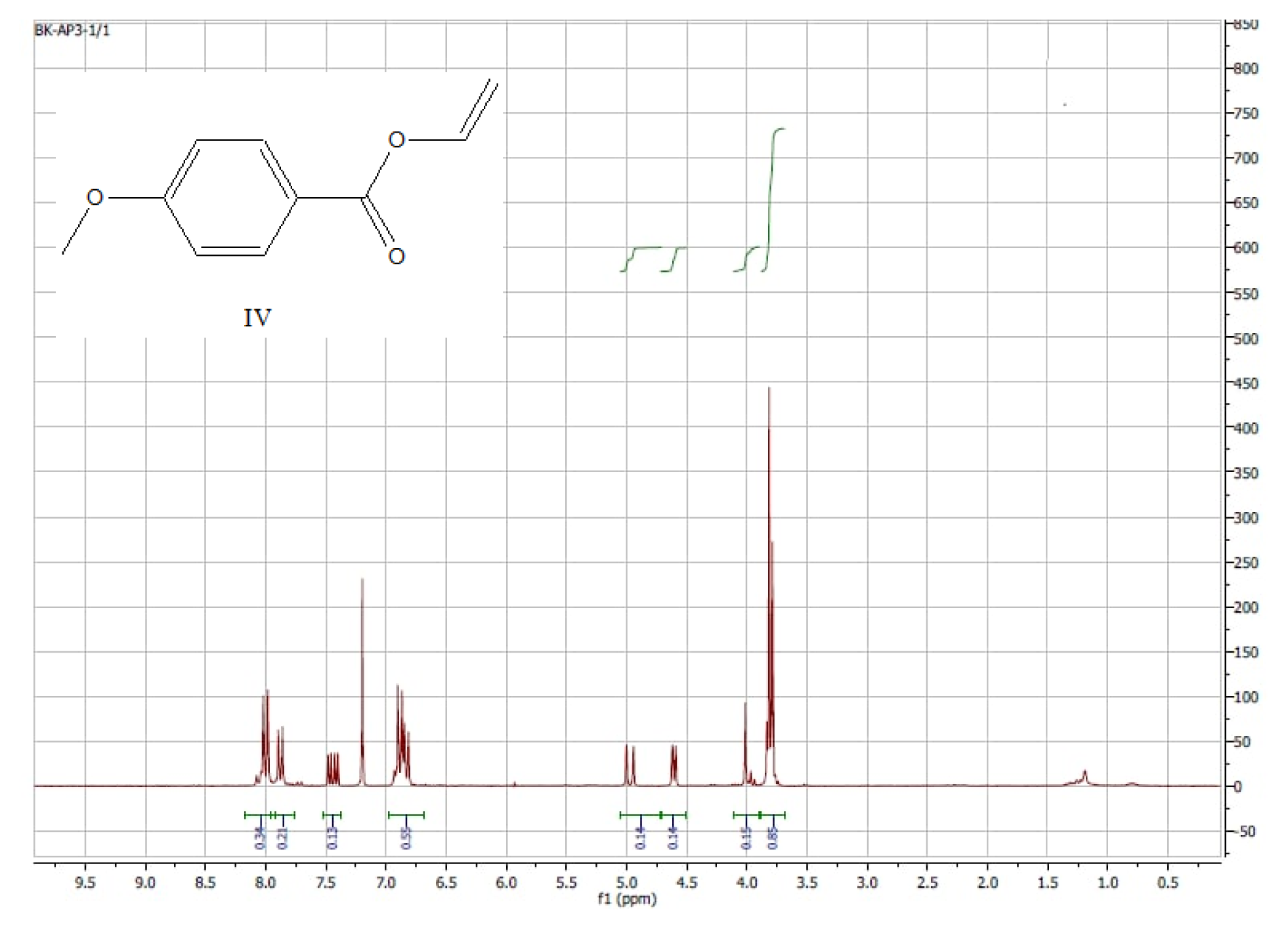

Supplement: Figure S11 — 1H- NMR spectrum of vinyl ester of 4-methoxybenzoic acid. [file tjc-49-05-520s11.tif]

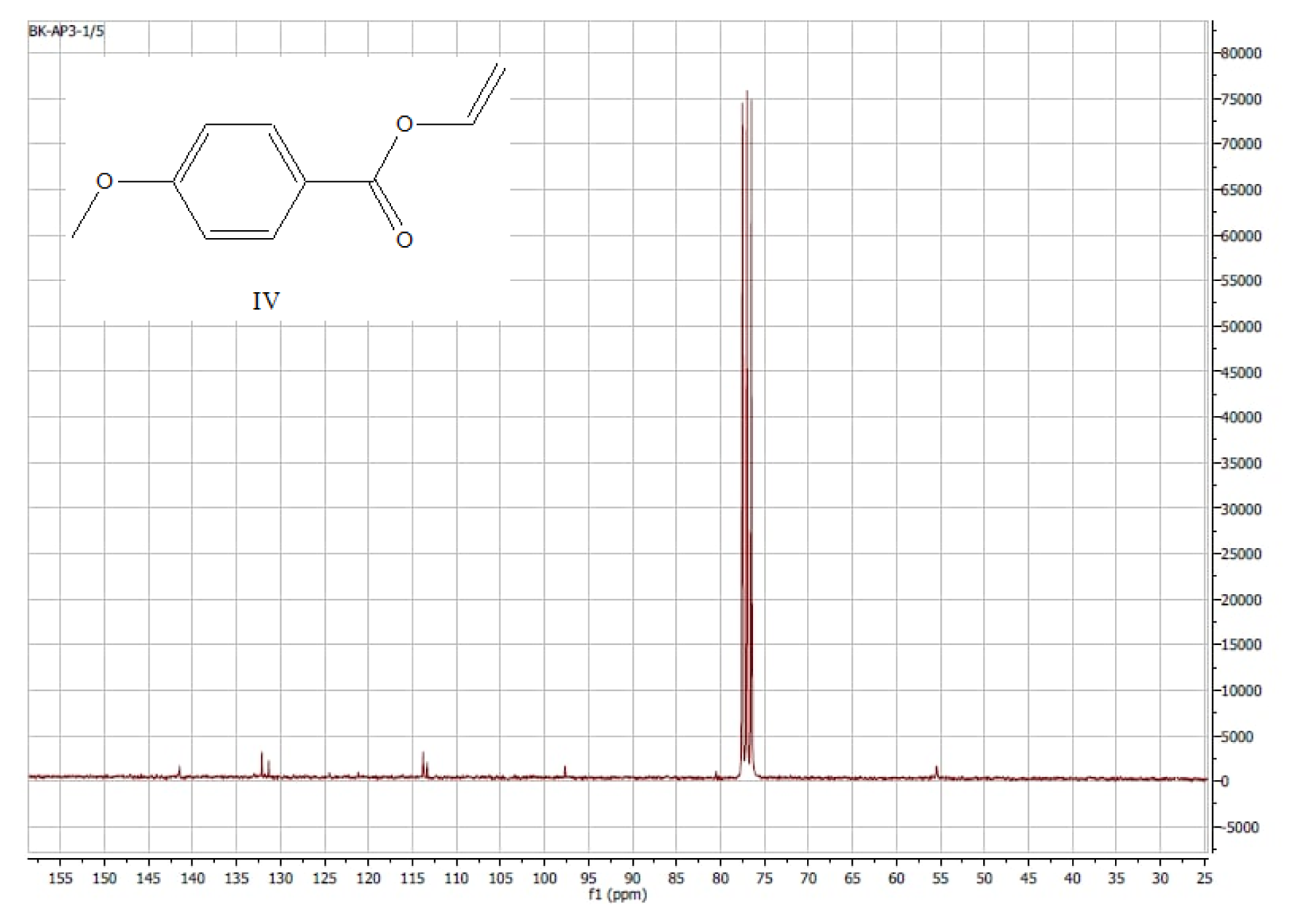

Supplement: Figure S12 — 13C- NMR spectrum of vinyl ester of 4-methoxybenzoic acid. [file tjc-49-05-520s12.tif]

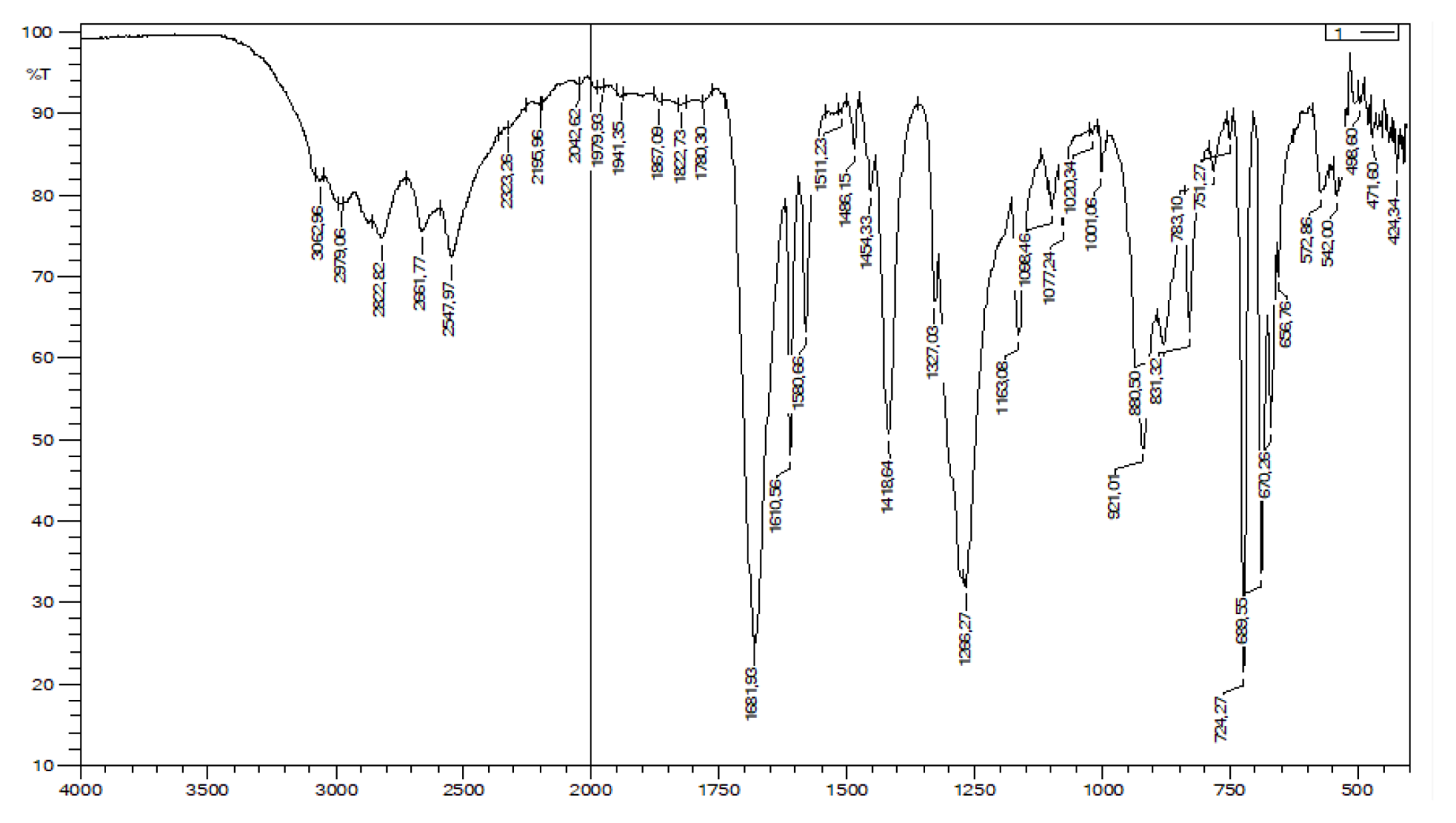

Supplement: Figure S13 — FTIR spectrum of vinyl ester of 4-methylbenzoic acid. [file tjc-49-05-520s13.tif]

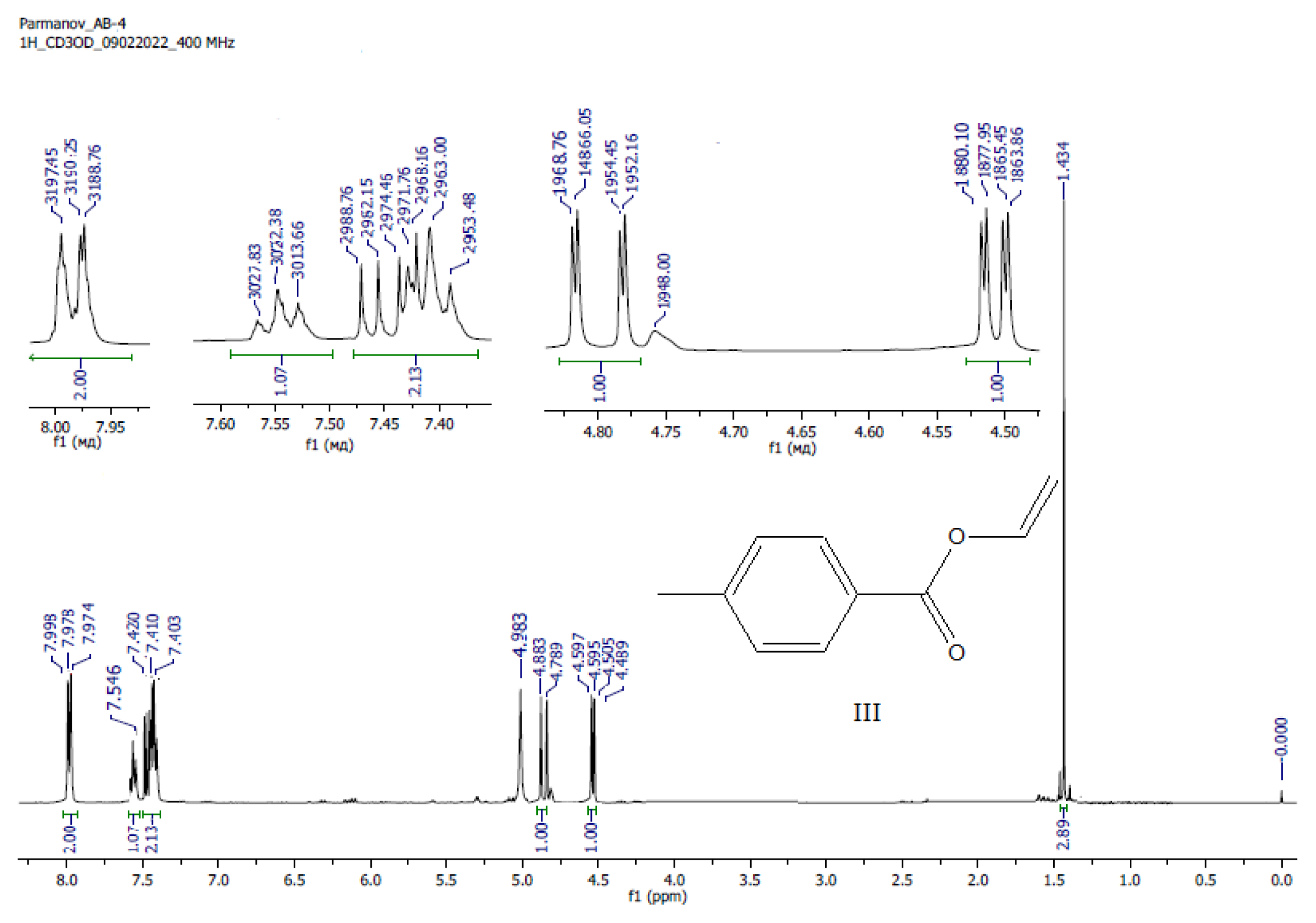

Supplement: Figure S14 — 1H- NMR spectrum of vinyl ester of 4-methylbenzoic acid. [file tjc-49-05-520s14.tif]

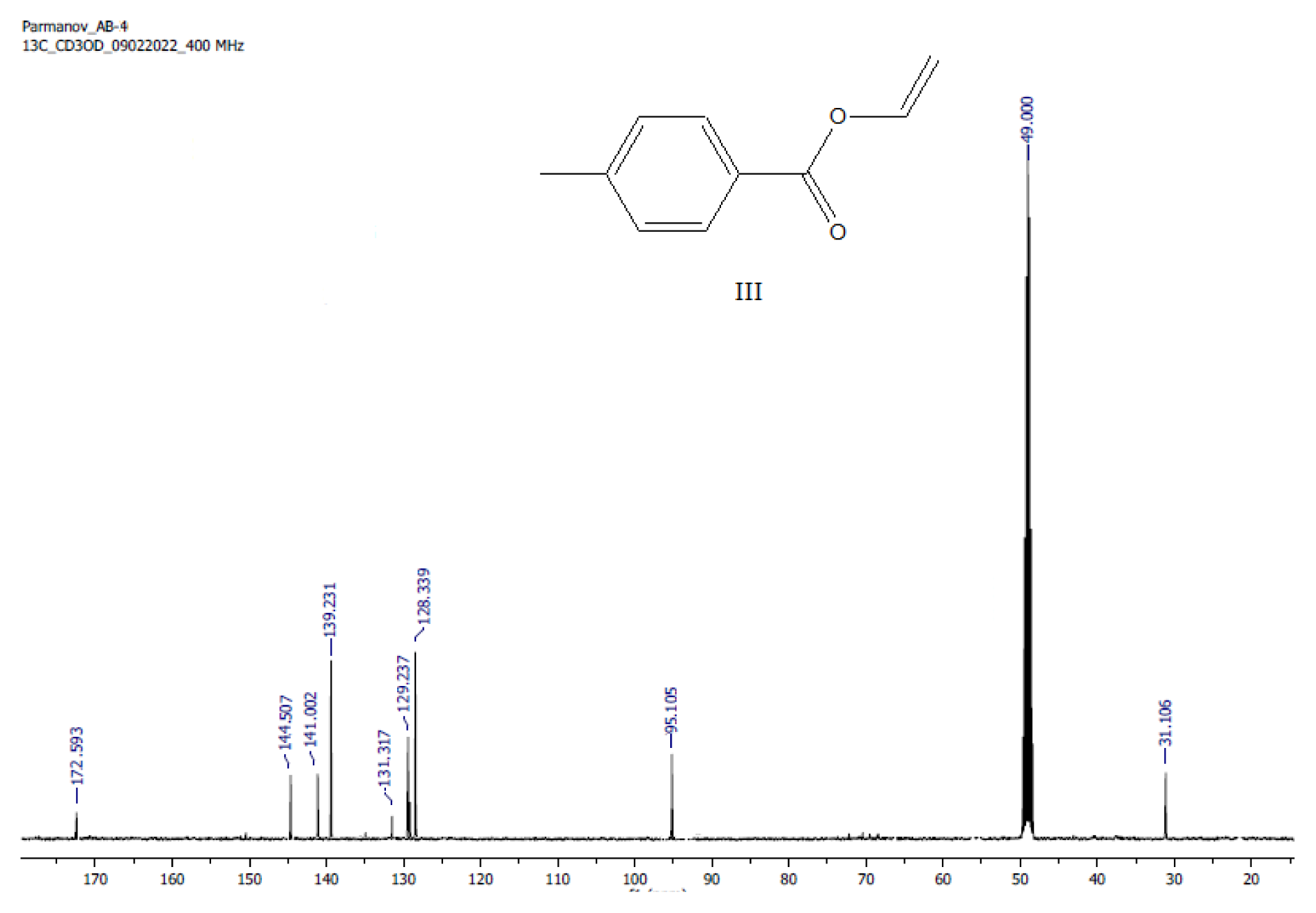

Supplement: Figure S15 — 13C NMR -spectrum of vinyl ester of 4-Methylbenzoic acid. [file tjc-49-05-520s15.tif]

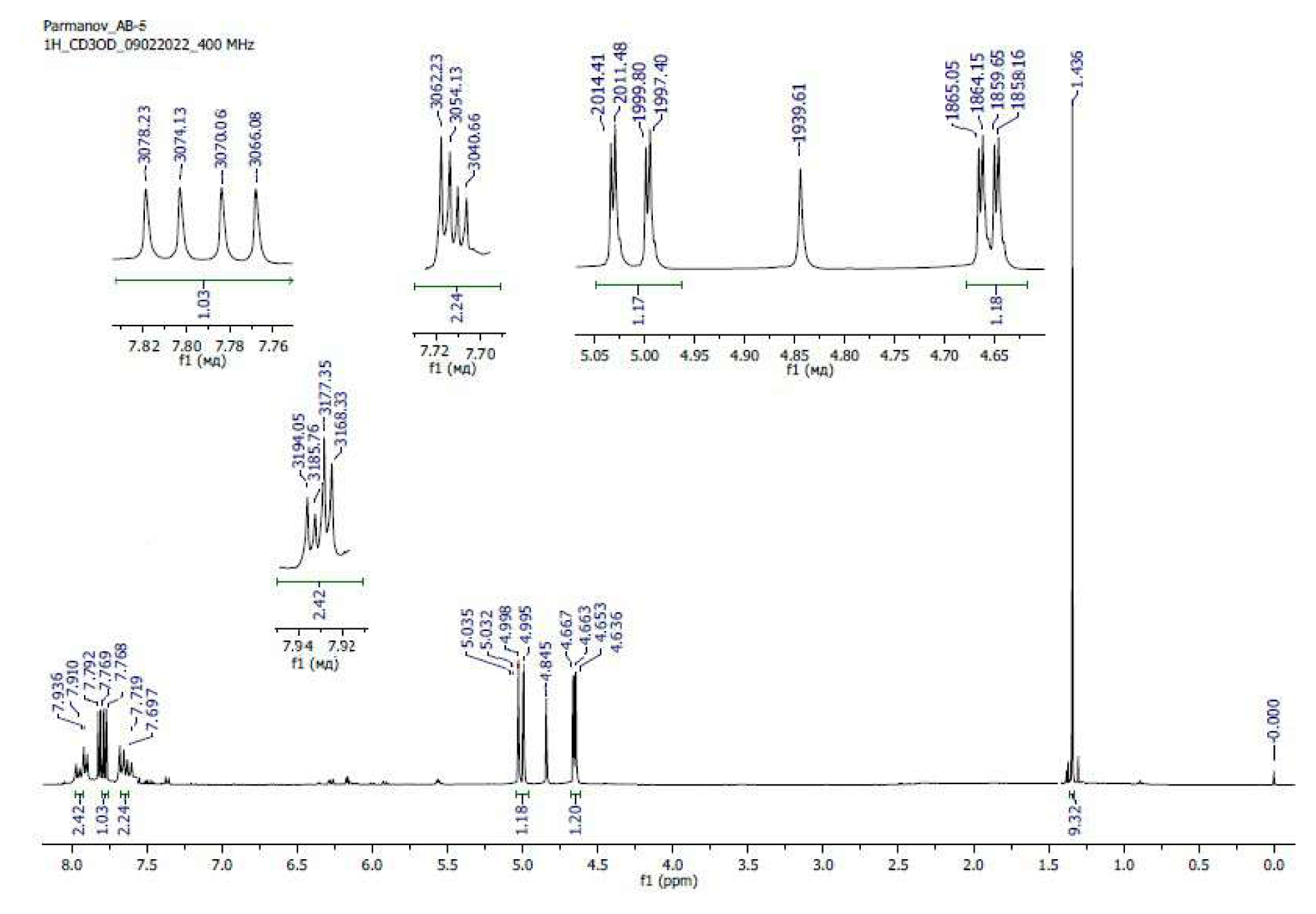

Supplement: Figure S16 — 1H-NMR spectrum of vinyl ester of 4-tert-butylbenzoic acid. [file tjc-49-05-520s16.tif]

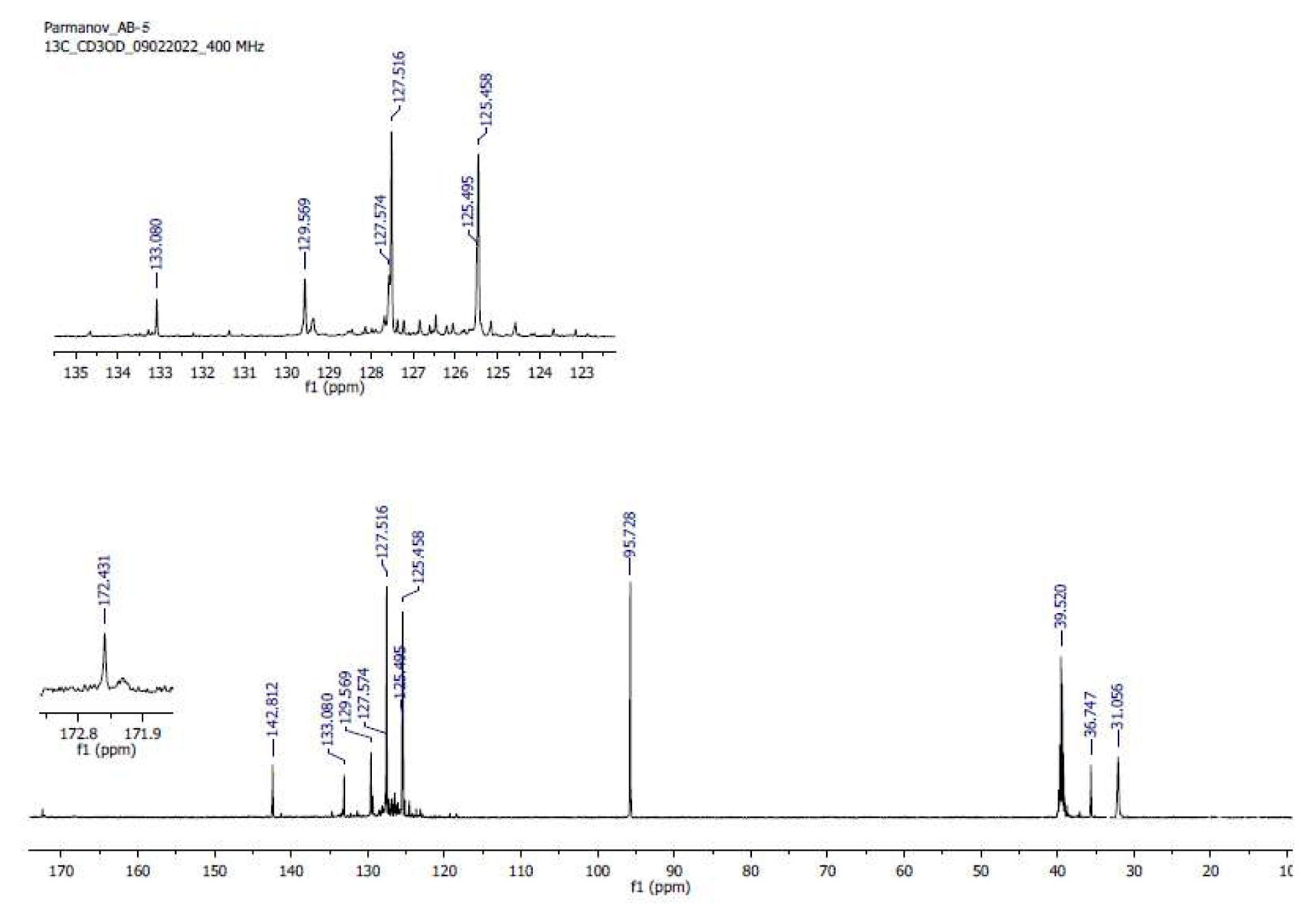

Supplement: Figure S17 — 13C NMR spectrum of vinyl ester of 4-tert-butylbenzoic acid. [file tjc-49-05-520s17.tif]

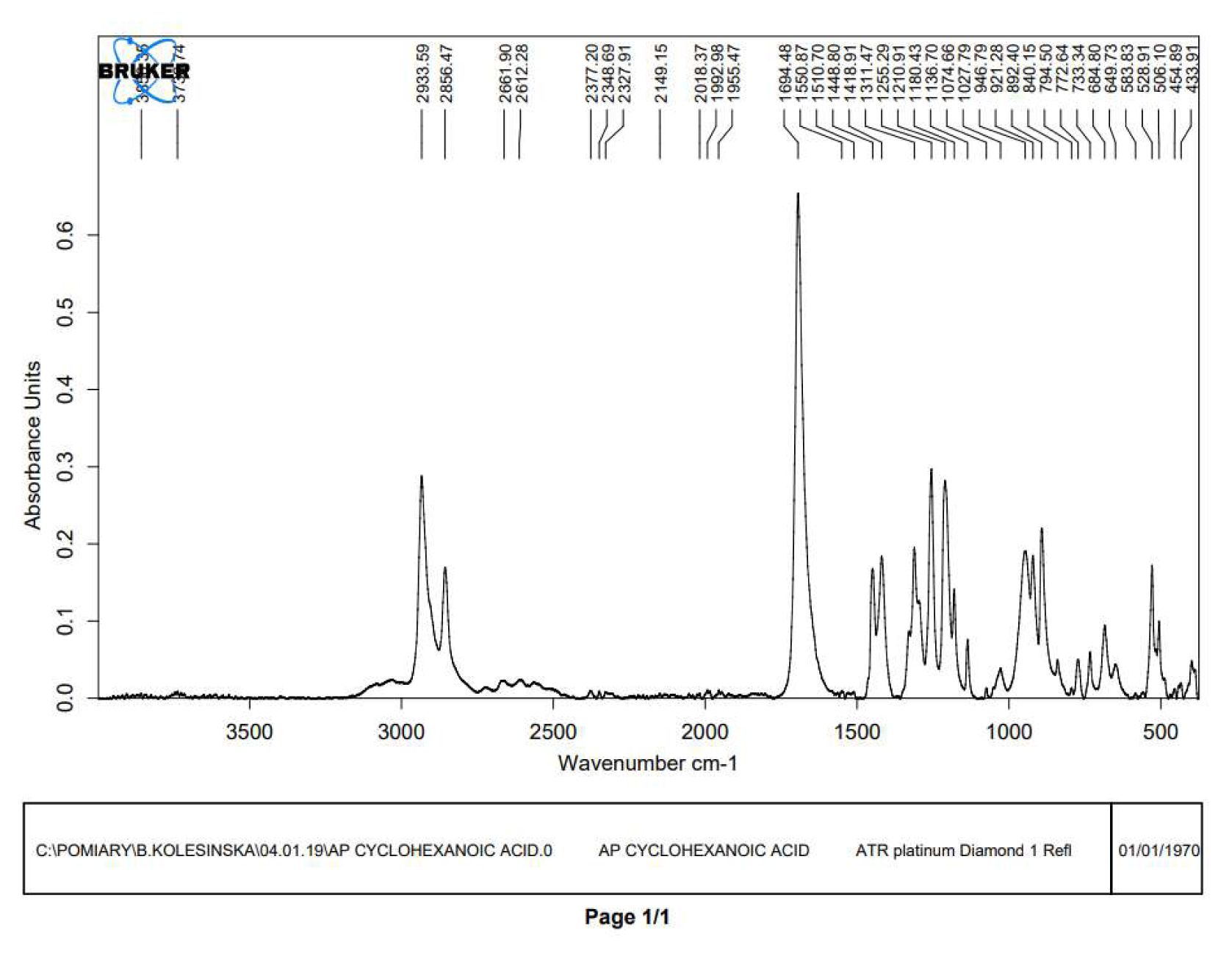

Supplement: Figure S18 — FTIR spectrum of vinyl ester of 4-chlorobenzoic acid. [file tjc-49-05-520s18.tif]

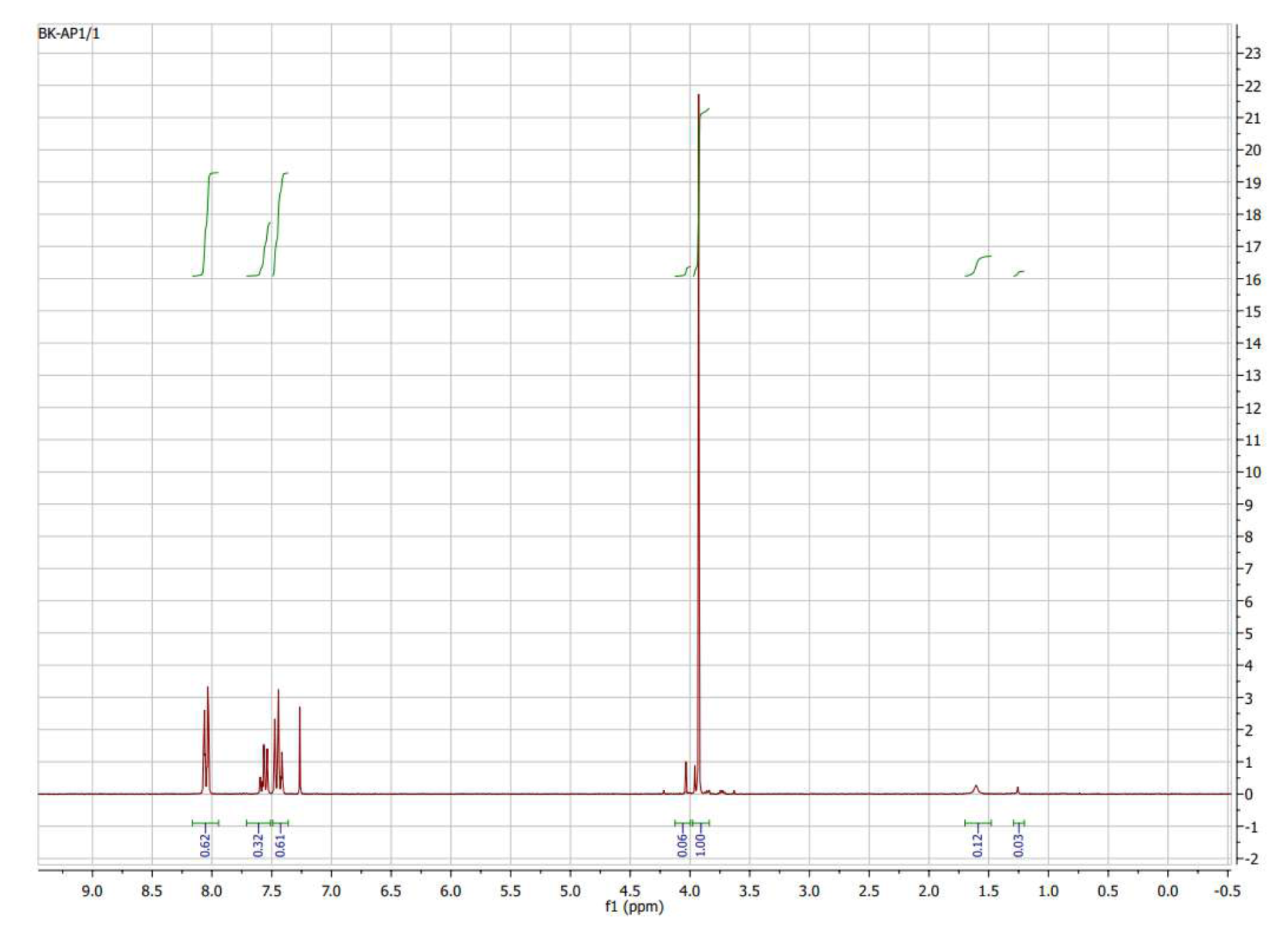

Supplement: Figure S19 — 1H-NMR spectrum of vinyl ester of 4-chlorobenzoic acid. [file tjc-49-05-520s19.tif]
